# Moderate Food Preferences and Depression with Grey Matter as a Potential Mediator: A Large-Scale Longitudinal Study

**DOI:** 10.3390/foods15172963

**Published:** 2026-08-24

**Authors:** Yi Cao, Zhaoying Li, Tong Wang, Tengxiao Guo, Dongfeng Zhang

**Affiliations:** 1 Biomedical Center, Qingdao University, No. 308 Ningxia Road, Qingdao 266021, China; guotengxiao@163.com; 2 Department of Epidemiology and Health Statistics, Public Health College, Qingdao University, No. 308 Ningxia Road, Qingdao 266021, China; wangtong4@qdu.edu.cn (T.W.); zhangdf1961@126.com (D.Z.)

**Keywords:** food preference, depression, brain structure, half-longitudinal design, cross-lagged analysis

## Abstract

**Background:** While stable dietary patterns and hedonic responses in food preferences are associated with depression, whether neurobiological mechanisms mediate this relationship remains unknown. This study therefore investigates the food preference–depression associations and their potential neurostructural mediation. **Methods:** Over 140,000 participants were included in a longitudinal study and cross-sectional. We employed Cox regression models to examine the relationships between food preferences and depression. Half-longitudinal mediation analysis and cross-lagged models estimated the mediating roles of brain grey matter and prospective relationships with depressive symptoms. **Results:** We found protective effects of moderate salty, bitter and spicy preferences against depression, especially in overweight and obese participants. Further, brain structure demonstrated widespread positive correlations with both food preferences and depressive symptoms. The mediation analysis identified volumes of peripheral cortical grey matter, ventricular cerebrospinal fluid, and grey and white matter as potential mediators in the relationships between salty preferences and depression. Of these, cross-lagged models revealed the distinct directional relationships between the volume of grey matter in the VI cerebellum (vermis)/lateral occipital cortex inferior division (right) and depression, indicating that their mutual influences are mediated through different neural mechanisms. **Conclusions:** Moderate, but not extreme, liking of specific tastes (spicy, bitter, or salty) reflects healthier mental states. The structure of brain grey matter may mediate the relationship between salty preference and depression; also, the cerebellum together with lateral occipital regions may potentially serve as potential emotional correlates. Further neurobiological investigations are needed to confirm this pathway and inform novel therapeutic strategies for depressive disorders.

## 1. Introduction

As a foremost contributor to global disability and reduced life-years, depression is characterised by anhedonia, pessimism, anxiety, and dysfunction in occupational and social domains [1]. By 2021, depression accounted for 357.44 million incident cases and 56.33 million disability-adjusted life-years globally, with approximately 727,000 suicides worldwide in the same year. Among all countries/regions, Uganda, Greenland, and Lesotho had the highest prevalence rates [2,3]. The etiopathogenesis of depression remains incompletely elucidated. It manifests as a complex interplay of multiple risk factors, including psychosocial stress, genetic predisposition, comorbid medical conditions, metabolic dysregulation, and suboptimal nutritional patterns [4,5,6,7,8,9,10,11].

Dietary factors play a crucial role in depression onset and symptom management [12,13,14,15]. Food preference scores assessing food patterns have demonstrated good correlations with actual food intake levels and a higher test–retest correlation without relying on memory, physical health status and social stress [16,17]. Methods for assessing food preferences vary among countries based on diverse cultures and economies. Commonly, Food Preference Questionnaires (FPQs) are designed based on predispositions for basic tastes, including bitter, salty, sweet, and sour [18,19]. They have been widely utilised in measuring the relationships with metabolic or mental disorders [20,21,22,23]. Food preferences reflect not only stable dietary patterns but also hedonic responses [24], both of which interact with brain structure. Neurobiological studies have linked these reward-related processes to specific brain regions, including the lateral hypothalamus, ventral pallidum, amygdala, and grey matter [25,26,27,28]. Genome-wide association studies (GWASs) further indicate that food preferences are associated with upregulated activity in the basal ganglia [29]. Structurally, higher fresh fruit consumption has been linked to greater grey matter volume in regions such as the right temporal occipital fusiform cortex, left postcentral gyrus, and right precentral gyrus—areas previously implicated in depression onset [30]. Similarly, moderate alcohol consumption was positively associated with medial orbitofrontal cortex thickness [31]. In depression, however, the translation from hedonic experience into motivated behaviour may break down due to impaired functional connectivity within reward-related circuits [32]. Complementing these findings, accumulating evidence highlights an important role for dietary nutrition to influence depression etiology through specific brain regions, including n-3 PUFA, polyphenols, vitamin D and B, and food patterns [33,34,35,36,37].

Recent studies have highlighted connections between food preference and depression; however, large-scale studies examining the specific role of taste-based food preferences in depression remain scarce. We hypothesised that specific food preferences may influence the development of depression and that this association is potentially mediated by brain structure. Here, we aim to evaluate the relationships between taste-liking preferences (bitter, salty, spicy, and sweet) and depression, and explore the potential mediated role of brain structure.

## 2. Methods

### 2.1. Study Populations

**Figure 1:**
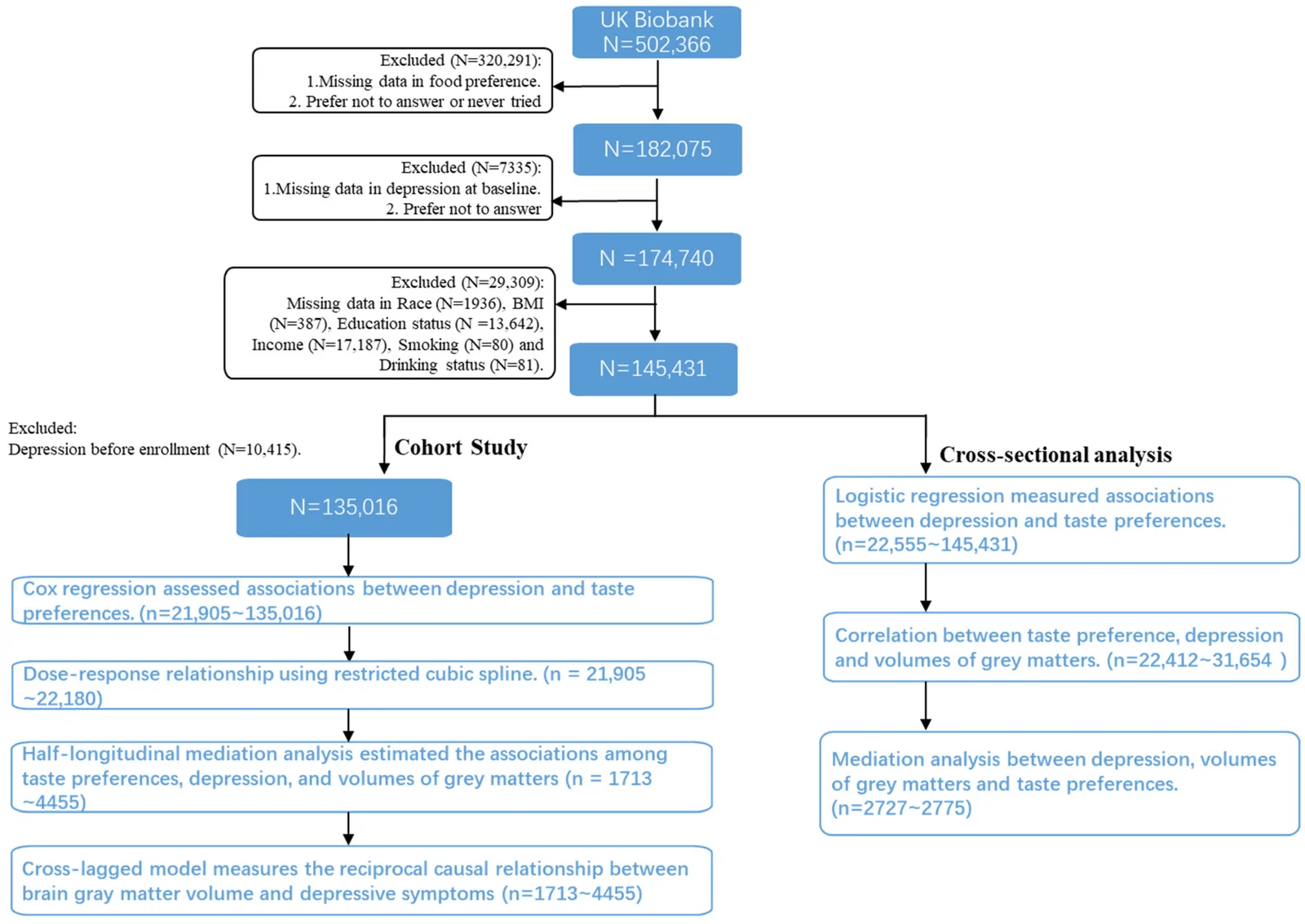
Flow diagram of sample selection and analytical procedures.

UK Biobank represents a large-scale biomedical database and research resource containing de-identified genomic, lifestyle and health information, as well as biological samples from over 500,000 UK participants. Baseline data were collected during 2006–2010 across 22 assessment centres through a standardised protocol consisting of five stages: (1) written consent; (2) touch screen questionnaires, i.e., detailed diet recall; (3) face-to-face interview with a study nurse; (4) measurements, i.e., hand grip, spirometry and bone density; and (5) sample collection of blood, urine and saliva. More detailed information about participants and quality control was published previously [38]. All participants have provided written informed consent. A flow diagram of sample selection and analytical procedures in the present study is presented in Figure 1.

### 2.2. Food Preferences Questionnaire

The food preferences questionnaire includes 150 items, comprising food items that reflect sensory preferences, foodstuff and health behaviour preferences. Over 180,000 participants completed the food preference web questionnaires. Participants were presented with a series of food and non-food items and asked to rate their liking for each item using a 9-point scale ranging from 1 (“extremely dislike”) to 9 (“extremely like”), and 5 represented neutral preference (“neither like nor dislike”). “Never tried” and “Do not wish to answer” were offered as additional options but were excluded from analysis due to the small sample of subjects. More details about food preferences can be found at https://biobank.ndph.ox.ac.uk/showcase/refer.cgi?id=25974 (accessed on 25 July 2025). The temporal stability of food preferences allows for their integration with the health and behavioural information collected at other time points. Here, predispositions for basic tastes of food preferences (bitter, salty, spicy, and sweet) were selected to evaluate the associations with depression.

### 2.3. Depression Outcome

Depressive symptoms were assessed using self-report of non-cancer illness and Patient Health Questionnaire-2 (PHQ-2) in cross-sectional study. The code for depression in non-cancer illness was 1286. In PHQ-2, the frequency of depressed mood and anhedonia over the past 14 days was inquired, with each item scored from 0 to 3 (from “not at all” to “nearly every day”). The total scores of PHQ-2 range from 0 to 6, and a cutoff of ≥3 points is defined as depression, with sensitivity of 83% and specificity of 92% for major depression [39].

In the cohort study, the 10th edition of the International Classification of Diseases (ICD-10) was applied into diagnosis of depression in hospital inpatient records in either the primary or secondary position. Depression cases were identified using ICD-10 codes F32 and F33. The end date for follow-up study period was 31 December 2022. Also, we acquired death data according to central registry. The endpoint was defined as the earliest of depression onset, death, or end of the follow-up period.

### 2.4. Brain Magnetic Resonance Imaging

Brain magnetic resonance imaging (MRI) was conducted using a standard Siemens Skyra 3T running VD13A SP4, with a standard Siemens 32-channel head coil. After preprocessing with aligning modalities and removing artefacts, the specific image modalities were optimised and combined into a pipeline through a fully automated processing pipeline primarily based around FSL software (version 5.0.10) [40]. The pipeline generates over 4300 imaging-derived phenotypes (IDPs), including summary measures, microstructural measures, and structural and functional connectivity metrics [41]. A total of 164 IDPs about volume of grey matter and white matter were selected as mediators in the relationships between food preference and depressive symptoms. All variables were subjected to standardisation by converting to Z-scores. The field IDs and descriptions of these IDPs are displayed in Supplementary Table S1. If a duplicated field name occurs, the normalised data for head size was adopted.

### 2.5. Covariates

The covariates in cross-sectional and cohort study included age, sex, ethnic background, body mass index (BMI), smoking status, drinking status, education levels, average total household income before tax, MET minutes per week for activity, hypertension, diabetes, and dyslipidemia (total cholesterol, total triglycerides, and HDL cholesterol). More details about definitions of covariates are given in Supplementary Methods.

### 2.6. Statistical Analysis

Baseline characteristics were summarised as mean (SD) for continuous variables and count (proportion) for categorical variables. Student’s *t* test was applied to compare the differences between groups for continuous variables and, for categorical variables, Chi-square test was used. Pearson and Spearman correlations and cross-sectional mediation were conducted with False Discovery Rate (FDR) adjusted (FDR < 0.05 was regarded as significant). We used binary logistic regression to estimate the associations between food preferences and depression in three models. To avoid biased estimates and extreme small sample size, level 5 was regarded as the reference group. Model 1 is a crude model, and Model 2 is adjusted for age and sex. We further adjusted ethnic background, BMI, smoking status, drinking status, education levels, average total household income before tax, MET minutes per week for activity, hypertension, diabetes, TG, total cholesterol, and HDL-C in Model 3.

Cox regression was conducted in the cohort study with the same variables adjusted as in three logistic regression models. *p* for trend test was calculated to estimate if there were linear associations between food preferences and depression. Then, we performed a restricted cubic spline (RCS) to describe these linear associations with 3 knots (5th, 50th, and 95th percentiles). Also, we performed subgroup analysis according to sex, age (<60 years old and ≥60 years old), and BMI (normal, overweight, and obesity). Furthermore, sensitive analysis was used to assess robustness of models excluding: (1) the participants with less than two-year follow-up and (2) the participants with extreme values of TG, glucose, blood pressure, and HDL-C levels (exceeding the range of 2.5–97.5%).

We used structural equation models in half-longitudinal mediation analysis to estimate the associations among food preferences, depression, and volumes of grey matter with two-wave follow-up data. Following Baron and Kenny’s stepwise approach and Sobel’s test [42,43], Cole and Maxwell extended mediation analysis from cross-sectional to longitudinal studies to design a practical compromise when full three-wave longitudinal data are not available [44]. We test the significance of the product of coefficient from X to M2 (a) and coefficient from M1 to Y2 (b), using bootstrap method (times = 1000). The 95% confidence intervals (CI) of a*b not containing 0 meant the significant mediating effects of brain volumes. r_XY1_, r_M2Y2_, r_XM1_, and r_M1Y1_ are the synchronous correlations between X, M and Y in the same periods; r_M_ and r_Y_ indicate autoregressions. Then, the cross-lagged model measures the reciprocal causal relationship between brain grey matter volume and depressive symptoms (2014+ and 2019+) with covariates adjusted. Harman’s single-factor test was used to assess common method bias. The following indices and corresponding standards were used to assess the fitting of the models: chi-square/degrees of freedom (CMIN/DF) < 5, root mean square error of approximation (RMSEA) and the standard root-mean-square (SRMR) < 0.06, and comparative fit index (CFI) and Tucker–Lewis index (TLI) > 0.9 [45]. Cross-sectional mediation analyses were performed using the *mediation* (4.5.1) package in R (version 4.4.3).

All of the statistical analysis was applied with R.4.4.3. *p*-values < 0.05 were considered as significant. The mediation analysis in cohort design was applied by *lavaan* (0.6.19) package.

## 3. Results

### 3.1. Baseline Characteristics

After selecting the participants according to inclusive and exclusive criteria, 145,431 participants were recruited in our analysis (Figure 1). According to the PHQ-2, the characteristics of UK biobank participants in the cross-sectional study are shown in Table S2. Baseline characteristics of UK Biobank participants in the cohort study are displayed in Table 1. The mean follow-up time is 9.4 years. Mean age of participants was 55.42 ± 7.68 years. During follow-up, 5510 (4.08%) participants were diagnosed with depression, with the highest incidence observed in the age group of 57.5–67.5 years. The differences between the groups of depression and controls occurs in most covariates, except sex.

### 3.2. The Associations Between Food Preferences and Depression/Depressive Symptoms: Cohort Study

Table 2 demonstrated the associations between food preferences and depression. After being fully adjusted in Model 3, moderately liking bitter food is a protective effect against depression, with HR (95%CI): 0.698 (0.547~0.891). As for salty foods, a moderate like (Levels 7 and 8) remained significantly associated with a 29% lower risk in Model 3 (HR (95%CI): 0.707 (0.526~0.951)). Liking spicy food (levels 6, 7, 8, and 9) was consistently associated with a significantly reduced risk of depression. This protective effect remained significant across Models 1–3 (HR: 0.651 (0.503~0.842) in level 9).

The *p* for trend test indicated linear relationships for liking bitter foods (*p* = 0.005) and spicy foods (*p* = 0.001). Then, the RCS demonstrated a significant decreased risk of depression from level 2 and transformed into a protective effect when the participants increased the extents of liking the bitter foods (Figure S1). Also, a similar trend was found in liking for spicy foods, which showed a significant decreased risk of depression from level 3 (Figure S2).

Furthermore, subgroup analysis was conducted stratified by sex, age, and BMI, respectively. The results of subgroup by sex and age were basically consistent with the main results (Tables S3–S6). The associations between liking for spicy foods and depression were mainly in overweight and obesity participants (Tables S7–S9). Meanwhile, sensitive analysis demonstrated the stability of the models after excluding the outliers of covariates (Tables S10–S14).

### 3.3. The Associations Between Food Preferences and Depressive Symptoms: Cross-Sectional Study

We observed similar results in the cross-sectional study (Table S15). In addition, several extreme food preferences, such as extreme dislike for bitter food or extreme like for salty and sweet food, were found to be risk factors to depression. Also, our analyses revealed significantly positive associations between most regional brain volumes and food preferences (Tables S16–S19), as well as between regional brain volumes and depressive symptoms (Table S20).

### 3.4. Half-Longitudinal Mediation Analysis

**Figure 2:**
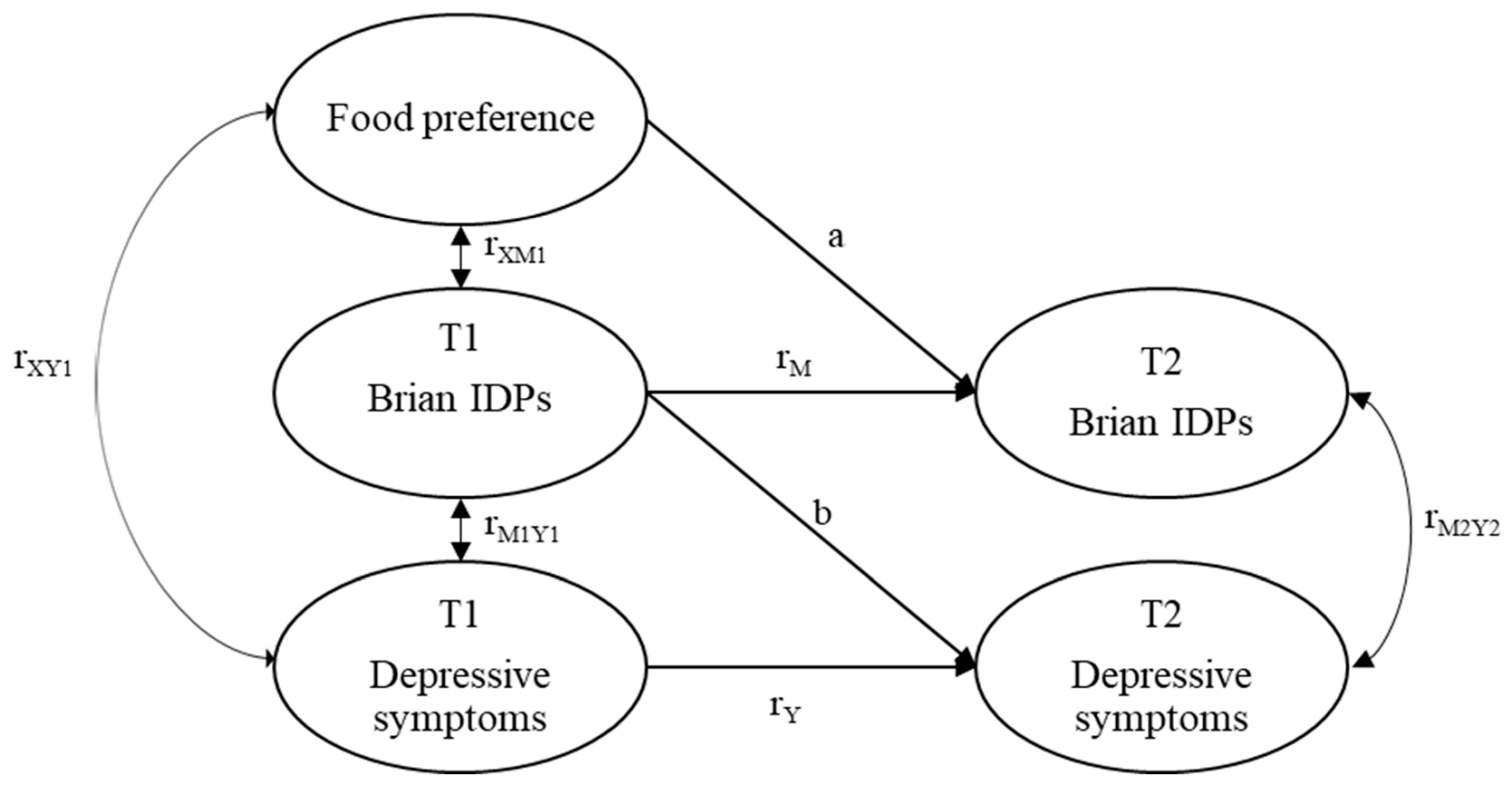
Illustration of structural equation models in half-longitudinal mediation analysis. r_XY1_, r_M2Y2_, r_XM1_, and r_M1Y1_ are the synchronous correlations between X, M and Y in the same periods; r_M_ and r_Y_ indicate autoregressions. We test the significance of the product of coefficient from X to M2 (a), coefficient from M1 to Y2 (b), and a*b. IDPs, imaging-derived phenotypes.

Harman’s single-factor test revealed that the first factor accounted for 31.86% of the variance, which is well below the critical threshold of 40%. Table 3 presents 11 well-fitted structural equation models in mediation analysis in half-longitudinal design. We observed the significant indirect effects of food preferences on depressive symptoms at T2 in mediation analysis (Figure 2); that is, the brain volume mediated the relationships between food preferences and depressive symptoms (Table 4). The volumes of peripheral cortical grey matter, ventricular cerebrospinal fluid, grey matter, and grey+white matter partly mediated the associations between liking for salty foods and depression. The volumes of grey matter in lateral occipital cortex, inferior division (right), VI cerebellum (vermis), and planum temporale (left) were mediators in the associations of liking for sweet foods and depression. Mediation results for all brain IDPs are shown in Table S21.

### 3.5. Mediation Analysis in Cross-Sectional Study

We conducted mediation analysis in the cross-sectional study and observed the volume of peripheral cortical grey matter and volume of grey matter were the potential mediators in the associations between liking for salty foods and depressive symptoms (Table S22). However, the mediated effects were not significant in further adjusted models (Table S23).

### 3.6. Cross-Lagged Model in Cohort Study

**Figure 3:**
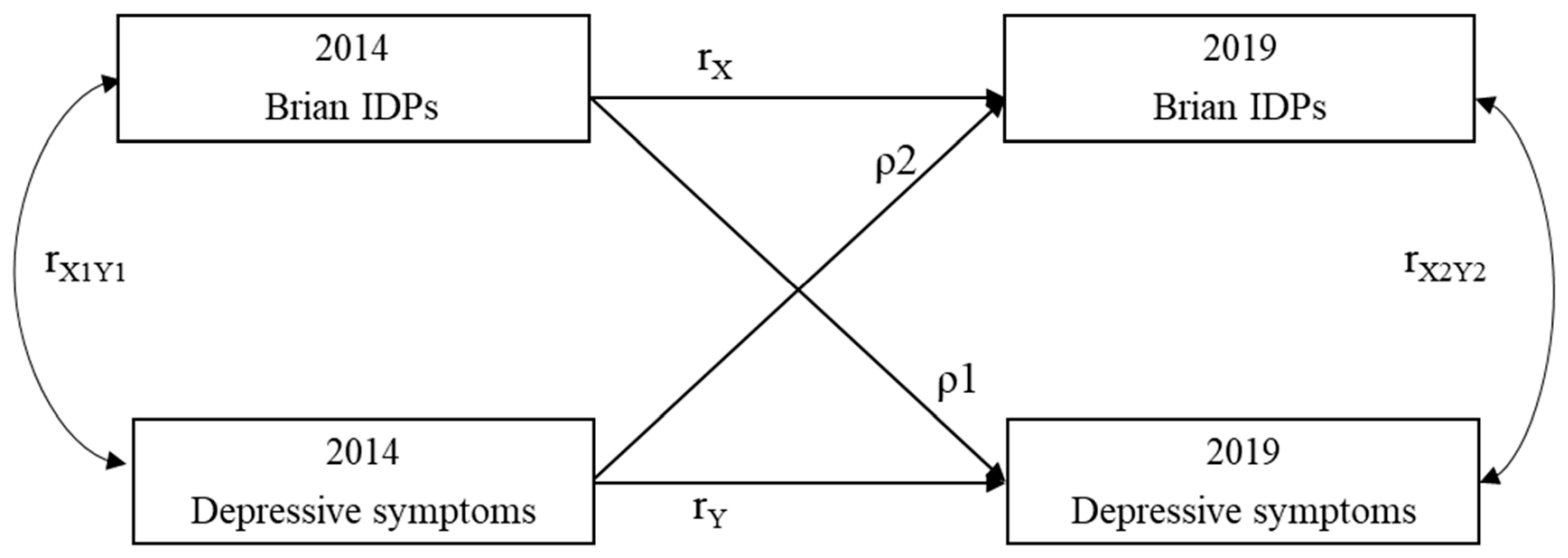
Illustration of cross-lagged model. ρ1 represents the path from 2014 brain IDPs to follow-up depression.; ρ2, the path from 2014 depression to follow-up brain IDPs in 2019; r_X1Y1_ and r_X2Y2_ are the synchronous correlations between X and Y in the same periods; r_X_ and r_Y_ indicate autoregressions. IDPs, imaging-derived phenotypes.

Then, illustration of two-wave cross-lagged models is displayed in Figure 3. And the models were used to test the prospective relationships between volumes of grey matter and depressive symptoms according to the results of Table 4. Since all models are saturated models, significant estimates are displayed in Table 5. Volume of grey matter in VI Cerebellum (vermis) predicted depressive symptoms at T2 and vice versa (ρ1 = 1.22 × 10^−4^, *p*-value = 0.014; ρ2 = −3.68, *p*-value = 0.047). Conversely, volume of grey matter in Lateral Occipital Cortex, inferior division (right), showed only a unidirectional predictive relationship with depressive symptoms at the next wave (ρ1 = −2.07 × 10^−5^, *p*-value = 0.028; ρ2 = −8.64, *p*-value = 0.203).

## 4. Discussion

Overall, the present study found moderate liking for bitter and salty food has a protective effect against depression; and a continuously decreasing risk of depression was found as the extent of liking spicy foods increased. Furthermore, we observed seven specific grey matter volumes mediated the relationships between food preferences and depressive symptoms. Among these, cross-lagged effects were significant bidirectionally between the volume of the cerebellar vermis (VI) and depressive symptoms, whereas the volume of the right inferior lateral occipital cortex exhibited a unidirectional effect on depressive symptoms.

Food preference has been reported to correlate with personality and to serve as a predictor of dietary behavior and even disease [46,47]. Bitter foods, such as mature hop bitter acids, are generally considered beneficial for improving cognitive function, attention, and mood state [48,49]. So far, the literature on food preference and depression is limited, and the conclusions regarding salty and spicy preferences remain inconsistent. Our results showed that a moderate liking for salty food was a protective factor against depression. In contrast, Ferraris et al. reported that a higher preference for salty foods was positively associated with severe depression and anxiety [50]. Previous research revealed a positive association between frequent spicy food consumption and depressive symptoms in adolescents [51,52]. While the other literature reported that spicy food had analgesic effect and elicited more positive emotions in older adults with meals [53,54]. Capsaicin is recognised as the beneficial bioactive ingredient for the central nervous system in spicy food [55]. Consistent with prior research on capsaicin, we also observed spicy preference serving as a protective factor against depression; however, we did not find the brain volumes as the mediators in their association.

Global evidence indicates a consistent positive association between high sugar consumption and increased risk of anxiety and depressive symptoms [56]. Although the result was not significant in the cohort study, we found that extreme liking for sweet food was a risk factor for depressive symptoms in the cross-sectional study. Moreover, this association was partially mediated by grey matter volume in the cerebellar vermis (lobule VI). Multiple studies have shown that grey matter volume is associated with depression [57,58,59]. The research of cerebellum has expanded from sensorimotor control to emotion, cognition, and reward processing. The cerebellum acts as a predictor of meal timing and reward availability, a real-time regulator of satiety via dopaminergic modulation, and an integrator of homeostatic and hedonic signals [60]. Bogoian et al. found a similar result that more severe general and somatic depressive symptoms were associated with larger grey matter volume in the cerebellum vermis VI, a region of the salience network with established alterations in depression [61]. The bidirectional cross-lagged relationships between cerebellar vermis grey matter volume and depressive symptoms further demonstrated opposing coefficient signs, indicating divergent neurobiological mechanisms underlying their mutual influence: cerebellar vermis at T1 might potentially initiate compensatory mechanisms to counteract depression, while the depression in T2 led to the degradation of cerebellar integrity at T2. Both VI cerebellum (vermis) and lateral occipital cortex are linked to visual processing, suggesting a potential pathway through which food preferences might influence depressive symptoms via visual mechanisms.

There are several strengths of the present study. Firstly, large-scale longitudinal design provided the causal relationships between food preferences and depression. Secondly, we evaluated the mediating effects of specific brain region volumes on the food preferences–depression associations through both half-longitudinal mediation and cross-lagged design, which enhanced the robustness of our results. Our findings elucidate potential neurobiological mechanisms underlying the relationship between food preferences and depression.

However, there are still limitations existing in the present study. First of all, the coefficients of direct effect in half-longitudinal models do not represent the same effect as the path in a three-wave design. Consequently, while we were able to test whether brain volumes function as a partial mediator, it was not feasible to accurately estimate the direct effect or quantify the proportion of mediation [44]. Additionally, this raises concerns regarding multiple testing correction based on *p*-values of proportion in the mediation analysis. Notably, our findings were exploratory and population-based and limited to structural features. The observed effect sizes should be interpreted in the context of risk factor identification. Whether this effect is clinically significant requires further research, such as intervention studies incorporating functional MRI to assess neural activity. Secondly, although we conducted mediation analysis in both longitudinal and cross-sectional studies, the sample size was substantially reduced with increasing follow-up waves, which may account for the non-significant findings in the cross-sectional mediation models. Thirdly, despite our findings were generally consistent across the cohort and cross-sectional studies, different definitions of outcomes being applied in these two designs may introduce bias. Next, while food preferences are quite stable over time in adults, a preference for a specific taste may correspond to the consumption of a wide variety of foods, each with different nutrient compositions, making it statistically complex and inefficient to isolate the effects of individual nutrients. Therefore, it is difficult to assess the effects of particular nutrients derived from dietary preferences on depression. Finally, given depression and food preferences are both influenced by genetic background and environment, it is necessary to investigate the intergenerational effects in the future.

## 5. Conclusions

A large-scale longitudinal study revealed that specific food preferences such as bitter salty or spicy were protective factors for depression. Characteristics of specific brain regions may represent a potential neurobiological mechanism through which dietary preferences influence depression onset. Beyond its established role, the VI cerebellum (vermis) or lateral occipital cortex inferior division (right) may potentially serve as potential emotional correlates in the link between food preferences and depression with distinct directional and mechanistic relationships. Our findings provided new insights into the potential mechanism among food preferences, brain structures and depression.

## Figures and Tables

**Table 1:** Characteristics of UK Biobank participants at baseline.

Traits	All Participants Free from Depression at Baseline (N = 135,016)	Participants with Depression During Follow-Up (N = 5510)	Participants Free of Depression During Follow-Up (N = 129,506)	*p*-Values *
**Age**	55.42 (7.68)	57.50 (7.66)	55.33 (7.66)	<0.001
**Sex**				0.63
Female	73,928 (54.75%)	2999 (54.43%)	70,929 (54.77%)	
Male	61,088 (45.25%)	2511 (45.57%)	58,577 (45.23%)	
**Ethnic background**				0.022
White	137,714 (97.32%)	5395 (97.91%)	126,011 (97.30%)	
Mixed	683 (0.51%)	25 (0.45%)	658 (0.51%)	
Asian or Asian British	1087 (0.80%)	34 (0.62%)	1053 (0.81%)	
Black or Black British	874 (0.65%)	31 (0.56%)	843 (0.65%)	
Chinese	299 (0.22%)	2 (0.03%)	297 (0.23%)	
Other ethnic group	667 (0.49%)	23 (0.42%)	644 (0.50%)	
**Average total household income**				<0.001
Less than 18,000	14,661 (10.86%)	1024 (18.58%)	13,637 (10.53%)	
18,000 to 30,999	30,006 (22.22%)	1556 (28.24%)	28,450 (21.97%)	
31,000 to 51,999	40,173 (29.75%)	1563 (28.37%)	38,610 (29.81%)	
52,000 to 100,000	38,321 (28.38%)	1107 (20.09%)	37,214 (28.74%)	
Greater than 100,000	11,855 (8.78%)	260 (4.72%)	11,595 (8.95%)	
**BMI**	26.58 (4.42)	27.90 (5.28)	26.52 (4.37)	<0.001
**Smoke**				<0.001
Never	79,569 (58.93%)	2700 (49.00%)	76,869 (59.36%)	
Previous	46,282 (34.27%)	2219 (40.27%)	44,063 (34.02%)	
Current	9165 (6.78%)	576 (10.54%)	8589 (6.63%)	
**Education**				<0.001
College or University degree	738,211 (50.76%)	2498 (45.34%)	66,035 (50.99%)	
A levels/AS levels or equivalent	19,418 (14.38%)	777 (14.10%)	18,641 (14.39%)	
O levels/GCSEs or equivalent	27,670 (20.49%)	1270 (23.05%)	26,400 (20.39%)	
CSEs or equivalent	5113 (3.79%)	228 (4.14%)	4885 (3.77%)	
NVQ or HND or HNC or equivalent	7236 (5.36%)	408 (7.40%)	6828 (5.27%)	
Other professional qualifications, e.g., nursing, teaching	7046 (5.22%)	329 (5.97%)	6717 (5.19%)	
**Drink**				<0.001
Never	3293 (2.44%)	169 (3.06%)	3124 (2.41%)	
Previous	3062 (2.27%)	217 (3.94%)	2845 (2.20%)	
Current	128,661 (95.29%)	5122 (92.95%)	123,539 (95.39%)	
**Physical activity**				<0.001
Walking for pleasure (not as a means of transport)	75,257 (55.74%)	2884 (52.34%)	72,373 (55.88%)	
Other exercises (e.g., swimming, cycling, keep fit, bowling)	11,952 (8.85%)	479 (8.69%)	11,473 (8.86%)	
Strenuous sports	869 (0.64%)	25 (0.45%)	844 (0.65%)	
Light DIY (e.g., pruning, watering the lawn)	4957 (3.67%)	289 (5.25%)	4668 (3.60%)	
Heavy DIY (e.g., weeding, lawn mowing, carpentry, digging)	1470 (1.09%)	82 (1.49%)	1388 (1.07%)	
**Systolic blood pressure**	135.94 (17.95)	137.64 (18.22)	135.87 (17.93)	<0.001
**Diastolic blood pressure**	81.71 (9.95)	82.27 (10.09)	81.69 (9.94)	<0.001
**Total Cholesterol**	4.61 (0.91)	4.52 (0.97)	4.61 (0.90)	<0.001
**HDL Cholesterol**	1.33 (0.33)	1.32 (0.34)	1.34 (0.33)	0.04
**Total Triglycerides**	1.25 (0.55)	1.29 (0.59)	1.24 (0.55)	0.002
**Glucose**	3.48 (0.99)	3.58 (1.33)	3.48 (0.97)	0.005

Mean (SD) was used in continuous variables; number (%) was used in categorised variables; *, comparison between participants with and without depression at the end of follow-up.

**Table 2:** Longitudinal associations of food preferences and depression with cox regression results.

Variable	Model 1 HR (95%CI)	Model 2 HR (95%CI)	Model 3 HR (95%CI)	*p* for Trend
**Liking for bitter foods**				0.005
1	1.322 (1.212~1.442) **	1.267 (1.160~1.383) **	1.210 (0.985~1.487)	
2	1.090 (0.984~1.208)	1.062 (0.959~1.178)	0.850 (0.658~1.098)	
3	1.034 (0.938~1.140)	1.023 (0.927~1.127)	0.996 (0.791~1.254)	
4	0.921 (0.835~1.016)	0.921 (0.835~1.016)	0.822 (0.647~1.043)	
5	Ref	Ref	Ref	
6	0.861 (0.784~0.946) **	0.884 (0.804~0.971) **	0.800 (0.637~1.005)	
7	0.839 (0.762~0.923) **	0.860 (0.781~0.947) **	0.698 (0.547~0.891) **	
8	0.754 (0.657~0.865) **	0.771 (0.672~0.885) **	0.886 (0.651~1.204)	
9	0.973 (0.810~1.170)	1.004 (0.835~1.207)	0.732 (0.441~1.215)	
**Liking for salty foods**				0.305
1	1.071 (0.963~1.191)	1.020 (0.916~1.134)	0.993 (0.780~1.266)	
2	0.956 (0.849~1.077)	0.919 (0.816~1.036)	0.895 (0.679~1.178)	
3	0.976 (0.872~1.092)	0.950 (0.849~1.063)	0.983 (0.763~1.265)	
4	0.909 (0.812~1.018)	0.907 (0.810~1.015)	1.053 (0.826~1.342)	
5	Ref	Ref	Ref	
6	0.863 (0.779~0.956) **	0.893 (0.806~0.989) *	0.895 (0.710~1.128)	
7	0.879 (0.794~0.973) *	0.924 (0.834~1.022)	0.932 (0.742~1.172)	
8	0.834 (0.736~0.944) **	0.889 (0.785~1.007)	0.707 (0.526~0.951) *	
9	0.965(0.834~1.117)	1.047 (0.904~1.212)	0.915 (0.649~1.290)	
**Liking for spicy foods**				0.001
1	1.208 (1.061~1.374) **	1.186 (1.042~1.349) **	0.959 (0.719~1.280)	
2	1.144 (0.986~1.329)	1.125 (0.969~1.307)	0.714 (0.495~1.031)	
3	0.998 (0.858~1.160)	0.991 (0.852~1.153)	0.713 (0.497~1.023)	
4	0.933 (0.806~1.081)	0.937 (0.809~1.085)	0.704 (0.502~0.989) *	
5	Ref	Ref	Ref	
6	0.857 (0.760~0.966) *	0.900 (0.798~1.015)	0.754 (0.577~0.984) *	
7	0.802 (0.717~0.898) **	0.852 (0.761~0.953) **	0.635 (0.494~0.816) **	
8	0.741 (0.660~0.831) **	0.802 (0.714~0.900) **	0.623 (0.482~0.806) **	
9	0.816 (0.727~0.915) **	0.915 (0.816~1.027)	0.651 (0.503~0.842) **	
**Liking for sweet foods**				0.222
1	1.042 (0.896~1.211)	0.994 (0.855~1.156)	1.139 (0.803~1.617)	
2	0.973 (0.848~1.116)	0.950 (0.828~1.090)	1.157 (0.842~1.591)	
3	0.822 (0.724~0.934) **	0.813 (0.715~0.923) **	1.004 (0.745~1.353)	
4	0.947 (0.843~1.065)	0.951 (0.846~1.070)	0.986 (0.738~1.318)	
5	Ref	Ref	Ref	
6	0.854 (0.776~0.940) **	0.886 (0.805~0.976) *	1.008 (0.794~1.280)	
7	0.875 (0.798~0.961) **	0.913 (0.832~1.002)	1.036 (0.822~1.305)	
8	0.937 (0.848~1.036)	0.986 (0.892~1.090)	1.105 (0.862~1.418)	
9	1.054 (0.950~1.170)	1.149 (1.035~1.275) **	1.250 (0.966~1.617)	

Model 1 is a crude model, and Model 2 is adjusted for age and sex. We further adjusted ethnic background, BMI, smoking status, drinking status, education levels, average total household income before tax, MET minutes per week for activity, hypertension, diabetes, TG, total cholesterol, and HDL-C in Model 3. *, *p*-value < 0.05. **, *p*-value < 0.001.

**Table 3:** Model fit indices for the structural equation models in half-longitudinal design (food preferences–brain IDPs–depressive symptoms).

X	Mediators	CHIS/DF	RMSEA	CFI	SRMR	TLI
Liking for salty foods	Volume of peripheral cortical grey matter	2.048	0.018	1.000	0.008	0.999
Liking for salty foods	Volume of ventricular cerebrospinal fluid	1.990	0.017	1.000	0.009	0.999
Liking for salty foods	Volume of grey matter	1.926	0.017	1.000	0.008	0.999
Liking for salty foods	Volume of brain, grey+white matter	1.902	0.017	1.000	0.008	0.999
Liking for sweet foods	Volume of grey matter in Lateral Occipital Cortex, inferior division (right)	4.757	0.032	0.999	0.011	0.997
Liking for sweet foods	Volume of grey matter in Planum Temporale (left)	4.863	0.032	0.999	0.011	0.997
Liking for sweet foods	Volume of grey matter in VI Cerebellum (vermis)	4.763	0.032	0.999	0.011	0.996

**Table 4:** Estimates of the associations among food preferences, brain structures, and depressive symptoms in mediation analysis.

X	Mediators	r_XY1_	r_M1Y1_	r_XM1_	r_M2Y2_	r_M_	r_Y_	a	b	ab (95%CI) ^a^
Liking for salty foods	Volume of peripheral cortical grey matter	1.47 × 10^−1^ **	4.96 × 10^−2^ **	1.78 × 10^−1^ **	−2.25 × 10^−3^	9.47 × 10^−1^ **	5.24 × 10^−1^ **	6.76 × 10^−3^ *	3.91 × 10^−2^ *	5.60 × 10^−5^ (8.95 × 10^−6^, 1.21 × 10^−4^)
Liking for salty foods	Volume of ventricular cerebrospinal fluid	1.47 × 10^−1^ **	−5.14 × 10^−2^ **	−1.61 × 10^−1^ **	2.68 × 10^−3^	9.79 × 10^−1^ **	5.24 × 10^−1^ **	−2.84 × 10^−3^ *	−3.93 × 10^−2^ **	2.00 × 10^−5^ (2.69 × 10^−6^, 4.20 × 10^−5^)
Liking for salty foods	Volume of grey matter	1.47 × 10^−1^ **	4.65 × 10^−2^ **	1.85 × 10^−1^ **	−2.98 × 10^−3^	9.40 × 10^−1^ **	5.23 × 10^−1^ **	6.19 × 10^−3^ *	4.57 × 10^−2^ **	5.92 × 10^−5^ (8.52 × 10^−6^, 1.24 × 10^−4^)
Liking for salty foods	Volume of brain, grey + white matter	1.47 × 10^−1^ **	4.14 × 10^−2^ *	1.85 × 10^−1^ **	−2.45 × 10^−3^	9.46 × 10^−1^ **	5.23 × 10^−1^ **	4.85 × 10^−3^ *	4.60 × 10^−2^ **	4.47 × 10^−5^ (5.28 × 10^−6^, 1.06 × 10^−4^)
Liking for sweet foods	Volume of grey matter in Lateral Occipital Cortex, inferior division (right)	6.79 × 10^−2^ *	3.57 × 10^−3^	−9.24 × 10^−3^	8.73 × 10^−4^	9.71 × 10^−1^ **	5.34 × 10^−1^ **	4.86 × 10^−3^ *	−2.75 × 10^−2^ *	−1.18 × 10^−4^ (−2.99 × 10^−4^, −9.21 × 10^−7^)
Liking for sweet foods	Volume of grey matter in VI Cerebellum (vermis)	6.79 × 10^−2^ *	3.47 × 10^−3^	4.89 × 10^−2^	3.05 × 10^−3^	9.30 × 10^−1^ **	5.34 × 10^−1^ **	1.01 × 10^−2^ **	3.27 × 10^−2^ *	−1.11 × 10^−4^ (−2.68 × 10^−4^, −1.05 × 10^−5^)
Liking for sweet foods	Volume of grey matter in Planum Temporale (left)	6.79 × 10^−2^ *	2.50 × 10^−2^	3.77 × 10^−2^	1.88 × 10^−3^	9.78 × 10^−1^ **	5.35 × 10^−1^ **	3.17 × 10^−3^ *	−2.61 × 10^−2^ *	3.42 × 10^−4^ (8.79 × 10^−5^, 7.31 × 10^−4^)

r_XY1_, r_M2Y2_, r_XM1_, and r_M1Y1_ are the synchronous correlations between X, M and Y in the same periods; r_M_ and r_Y_ indicate autoregressions. ^a^, The 95% confidence intervals (CI) of ab not containing 0 meant the significant mediating effects of brain volumes. *, *p*-value < 0.05; **, *p*-value < 0.001.

**Table 5:** Cross-lagged coefficients for brain volumes and depressive symptoms.

Brain Volumes	ρ1	*p*-Value	ρ2	*p*-Value
Volume of grey matter in VI Cerebellum (vermis)	1.22 × 10^−4^	0.014	−3.68	0.047
Volume of grey matter in Lateral Occipital Cortex, inferior division (right)	−2.07 × 10^−5^	0.028	−8.64	0.203
Volume of grey matter in Planum Temporale (left)	−3.01 × 10^−5^	0.230	0.41	0.820
Volume of peripheral cortical grey matter (normalised for head size)	−4.74 × 10^−7^	0.279	40.46	0.868
Volume of grey matter (normalised for head size)	−1.90 × 10^−7^	0.620	49.36	0.865
Volume of ventricular cerebrospinal fluid (normalised for head size)	1.81 × 10^−7^	0.813	−3.46	0.949
Volume of brain, grey+white matter (normalised for head size)	−5.25 × 10^−9^	0.980	−159.04	0.718

## Data Availability

The datasets in the current study are available at UK Biobank website: https://www.ukbiobank.ac.uk/ (accessed on 25 July 2025).

## Supplementary Materials

The following supporting information can be downloaded at: https://www.mdpi.com/article/10.3390/foods15172963/s1, Supplementary Materials contain methods (information of covariates) and results: Figure S1: Restricted cubic spline for the risk to the extent of liking for bitter food. The associations were adjusted for age, sex, ethnic background, BMI, smoking status, drinking status, education levels, average total household income before tax, MET minutes per week for activity, hypertension, diabetes, TG, total cholesterol, and HDL-C. The solid and dashed lines represent the estimated HRs (Hazard Ratio) and their 95% confidence intervals; Figure S2: Restricted cubic spline for the risk to the extent of liking for spicy food. The associations were adjusted for age, sex, ethnic background, BMI, smoking status, drinking status, education levels, average total household income before tax, MET minutes per week for activity, hypertension, diabetes, TG, total cholesterol, and HDL-C. The solid and dashed lines represent the estimated HRs (Hazard Ratio) and their 95% confidence intervals; Table S1: The basic information of brain imaging-derived phenotypes (IDPs); Table S2: Characteristics of UK Biobank Participants with or without depressive symptoms in cross-sectional study; Table S3: Cox Regression Results for food preferences and depression (HR, 95%CI, and Significance) in female participants; Table S4: Cox Regression Results for food preferences and depression (HR, 95%CI, and Significance) in male participants; Table S5: Cox Regression Results for food preferences and depression (HR, 95%CI, and Significance) in <60 year-old participants; Table S6: Cox Regression Results for food preferences and depression (HR, 95%CI, and Significance) in ≥60 year-old participants; Table S7: Cox Regression Results for food preferences and depression (HR, 95%CI, and Significance) in participants with normal BMI; Table S8: Cox Regression Results for food preferences and depression (HR, 95%CI, and Significance) in overweight participants; Table S9: Cox Regression Results for food preferences and depression (HR, 95%CI, and Significance) in obesity participants; Table S10: Sensitive analysis of cox regression results for food preferences and depression (HR, 95%CI, and Significance) without <2-year follow-up; Table S11: Sensitive analysis of cox regression results for food preferences and depression (HR, 95%CI, and Significance) with excluding extreme glucose levels; Table S12: Sensitive analysis of cox regression results for food preferences and depression (HR, 95%CI, and Significance) with excluding extreme TG levels; Table S13: Sensitive analysis of cox regression results for food preferences and depression (HR, 95%CI, and Significance) with excluding extreme blood pressures levels; Table S14: Sensitive analysis of cox regression results for food preferences and depression (HR, 95%CI, and Significance) with excluding extreme blood pressures levels; Table S15: Logistic regression results for food preferences and depressive symptoms; Table S16: The correlations between bitter preference and brain structures; Table S17: The correlations between salty preference and brain structures; Table S18: The correlations between spicy preference and brain structures; Table S19: The correlations between sweet preference and brain structures; Table S20: The correlations between depressive symptoms and brain structures; Table S21: All model fit indices for the structural equation models in half-longitudinal design (food preferences-brain sructures-depressive symptoms); Table S22: Mediating effects of brain structures on the associations between food preferences and depressive symptoms with age and sex adjusted in cross-sectional study; Table S23: Mediating effects of brain structures on the associations between food preferences and depressive symptoms with full covariates adjusted in cross-sectional study.

## Abbreviations

BMI	Body Mass Index
CFI	Comparative Fit Index
CMIN/DF	Chi-square/degrees of freedom
FFQs	Food Frequency Questionnaires
GWAS	Genome-Wide Association Studies
ICD-10	International Classification of Diseases, 10th Revision
IDPs	Imaging-Derived Phenotypes
PHQ-2	Patient Health Questionnaire-2
RCS	Restricted Cubic Spline
RMSEA	Root Mean Square Error of Approximation
SRMR	Standard Root-Mean-Square
TLI	Tucker-Lewis Index
