# Supplementary material for: Moderate Food Preferences and Depression with Grey Matter as a Potential Mediator: A Large-Scale Longitudinal Study
Source: Foods. 2026 Aug 24;15(17):2963. doi: 10.3390/foods15172963 (PMC13564540; doi:10.3390/foods15172963)
Supplement: Supplementary file 1 [file foods-15-02963-s001.zip › Supplementary Information.pdf]

Supplementary Materials

Moderate food preferences and depression with grey matter as a potential mediator: a large-scale longitudinal study

Yi Cao[1*†], Zhaoyig Li[1,2*†], Tong Wang[2], Tengxiao Guo[1], Dongfeng Zhang[2]

1: Biomedical Center, Qingdao University, No. 308 Ningxia Road, Qingdao 266021, China;

2: Department of Epidemiology and Health Statistics, Public Health College, Qingdao University, No. 308 Ningxia Road, Qingdao 266021, China;

*: Correspondence: qddxcaoyi@qdu.edu.cn; lizy0130@126.com.

†: These authors contributed equally to this work.

Key words: Depression; food preference; brain structure; half-longitudinal design; mediation analysis.

Contents

Supplementary methods **3**
Covariates: 3
Table S1. The basic information of brain imaging-derived phenotypes (IDPs). 3
Supplementary Results **8**
Table S2. Characteristics of UK Biobank Participants with or without depressive symptoms in cross-sectional study. 8
Table S3. Cox Regression Results for food preferences and depression (HR, 95%CI, and Significance) in female participants. 9
Table S4. Cox Regression Results for food preferences and depression (HR, 95%CI, and Significance) in male participants. 10
Table S5. Cox Regression Results for food preferences and depression (HR, 95%CI, and Significance) in <60 year-old participants. 12
Table S6. Cox Regression Results for food preferences and depression (HR, 95%CI, and Significance) in ≥60 year-old participants. 13
Table S7. Cox Regression Results for food preferences and depression (HR, 95%CI, and Significance) in participants with normal BMI. 14
Table S8. Cox Regression Results for food preferences and depression (HR, 95%CI, and Significance) in overweight participants. 15
Table S9. Cox Regression Results for food preferences and depression (HR, 95%CI, and Significance) in obesity participants. 17
Table S10. Sensitive analysis of cox regression results for food preferences and depression (HR, 95%CI, and Significance) without <2-year follow-up. 18
Table S11. Sensitive analysis of cox regression results for food preferences and depression (HR, 95%CI, and Significance) with excluding extreme glucose levels. 19
Table S12. Sensitive analysis of cox regression results for food preferences and depression (HR, 95%CI, and Significance) with excluding extreme TG levels. 20
Table S13. Sensitive analysis of cox regression results for food preferences and depression (HR, 95%CI, and Significance) with excluding extreme blood pressures levels. 22
Table S14. Sensitive analysis of cox regression results for food preferences and depression (HR, 95%CI, and Significance) with excluding extreme HDL-C levels. 23
Table S15. Logistic regression results for food preferences and depressive symptoms. 24
Table S16-23 were showed in supplementary table.xlsx 25
Figure S1. Restricted cubic spline for the risk to the extent of liking for bitter food. 26
Figure S2. Restricted cubic spline for the risk to the extent of liking for spicy food. 27

Supplementary methods

Covariates:

A. Age: The age was calculated based on the date of attendance at the assessment center and the date of birth.
B. Sex: Male and female.
C. Ethnic background: White; Mixed; Asian or Asian British; Black or black British; and other.
D. Body mass index (BMI) is based on the formula (weight (kg) / height (m^2)).
E. Smoking status: Never; Previous; Current.
F. Drinking status: Never; Previous; Current.
G. Education levels: College or University degree; A levels/AS levels or equivalent; O levels/GCSEs or equivalent; CSEs or equivalent; NVQ or HND or HNC or equivalent; Other professional qualifications eg: nursing; teaching; None of the above; Prefer not to answer.
H. MET minutes per week for activity: MET minutes per week for walking; MET minutes per week for moderate activity; MET minutes per week for vigorous activity.
I. Average total household income before tax: Less than 18;000; 18;000 to 30;999; 31;000 to 51;999; 52;000 to 100;000; Greater than 100;000.
J. Hypertension: hypertension was recognized according to any of the following criteria: Mean systolic blood pressure >=140 mmHg, or diastolic blood pressure >=90 mmHg;
K. Diabetes: Glucose from Nightingale Health data >= 7mmol/L; Take insulin regularly; Take diabetes lowering medicine regularly;
L. Dyslipidemia: Total Cholesterol from Nightingale Health Data >=6.2 mmol/L; or Total triglycerides from Nightingale Health data >=2.3 mmol/L; or HDL cholesterol from Nightingale Health data <=1.0mmol/L.

Table S1. The basic information of brain imaging-derived phenotypes (IDPs).

Field ID	Descriptions
25001	Volume of peripheral cortical grey matter (normalised for head size)
25002	Volume of peripheral cortical grey matter
25003	Volume of ventricular cerebrospinal fluid (normalised for head size)
25004	Volume of ventricular cerebrospinal fluid
25005	Volume of grey matter (normalised for head size)
25006	Volume of grey matter
25007	Volume of white matter (normalised for head size)
25008	Volume of white matter

25009	Volume of brain, grey+white matter (normalised for head size)
25010	Volume of brain, grey+white matter
25011	Volume of thalamus (left)
25012	Volume of thalamus (right)
25013	Volume of caudate (left)
25014	Volume of caudate (right)
25015	Volume of putamen (left)
25016	Volume of putamen (right)
25017	Volume of pallidum (left)
25018	Volume of pallidum (right)
25019	Volume of hippocampus (left)
25020	Volume of hippocampus (right)
25021	Volume of amygdala (left)
25022	Volume of amygdala (right)
25023	Volume of accumbens (left)
25024	Volume of accumbens (right)
25025	Volume of brain stem + 4th ventricle
25782	Volume of grey matter in Frontal Pole (left)
25783	Volume of grey matter in Frontal Pole (right)
25784	Volume of grey matter in Insular Cortex (left)
25785	Volume of grey matter in Insular Cortex (right)
25786	Volume of grey matter in Superior Frontal Gyrus (left)
25787	Volume of grey matter in Superior Frontal Gyrus (right)
25788	Volume of grey matter in Middle Frontal Gyrus (left)
25789	Volume of grey matter in Middle Frontal Gyrus (right)
25790	Volume of grey matter in Inferior Frontal Gyrus, pars triangularis (left)
25791	Volume of grey matter in Inferior Frontal Gyrus, pars triangularis (right)
25792	Volume of grey matter in Inferior Frontal Gyrus, pars opercularis (left)
25793	Volume of grey matter in Inferior Frontal Gyrus, pars opercularis (right)
25794	Volume of grey matter in Precentral Gyrus (left)
25795	Volume of grey matter in Precentral Gyrus (right)
25796	Volume of grey matter in Temporal Pole (left)
25797	Volume of grey matter in Temporal Pole (right)
25798	Volume of grey matter in Superior Temporal Gyrus, anterior division (left)
25799	Volume of grey matter in Superior Temporal Gyrus, anterior division (right)
25800	Volume of grey matter in Superior Temporal Gyrus, posterior division (left)
25801	Volume of grey matter in Superior Temporal Gyrus, posterior division (right)
25802	Volume of grey matter in Middle Temporal Gyrus, anterior division (left)
25803	Volume of grey matter in Middle Temporal Gyrus, anterior division (right)
25804	Volume of grey matter in Middle Temporal Gyrus, posterior division (left)
25805	Volume of grey matter in Middle Temporal Gyrus, posterior division (right)
25806	Volume of grey matter in Middle Temporal Gyrus, temporooccipital part (left)
25807	Volume of grey matter in Middle Temporal Gyrus, temporooccipital part (right)
25808	Volume of grey matter in Inferior Temporal Gyrus, anterior division (left)

25809	Volume of grey matter in Inferior Temporal Gyrus, anterior division (right)
25810	Volume of grey matter in Inferior Temporal Gyrus, posterior division (left)
25811	Volume of grey matter in Inferior Temporal Gyrus, posterior division (right)
25812	Volume of grey matter in Inferior Temporal Gyrus, temporooccipital part (left)
25813	Volume of grey matter in Inferior Temporal Gyrus, temporooccipital part (right)
25814	Volume of grey matter in Postcentral Gyrus (left)
25815	Volume of grey matter in Postcentral Gyrus (right)
25816	Volume of grey matter in Superior Parietal Lobule (left)
25817	Volume of grey matter in Superior Parietal Lobule (right)
25818	Volume of grey matter in Supramarginal Gyrus, anterior division (left)
25819	Volume of grey matter in Supramarginal Gyrus, anterior division (right)
25820	Volume of grey matter in Supramarginal Gyrus, posterior division (left)
25821	Volume of grey matter in Supramarginal Gyrus, posterior division (right)
25822	Volume of grey matter in Angular Gyrus (left)
25823	Volume of grey matter in Angular Gyrus (right)
25824	Volume of grey matter in Lateral Occipital Cortex, superior division (left)
25825	Volume of grey matter in Lateral Occipital Cortex, superior division (right)
25826	Volume of grey matter in Lateral Occipital Cortex, inferior division (left)
25827	Volume of grey matter in Lateral Occipital Cortex, inferior division (right)
25828	Volume of grey matter in Intracalcarine Cortex (left)
25829	Volume of grey matter in Intracalcarine Cortex (right)
25830	Volume of grey matter in Frontal Medial Cortex (left)
25831	Volume of grey matter in Frontal Medial Cortex (right)
25832	Volume of grey matter in Juxtapositional Lobule Cortex (formerly Supplementary Motor Cortex) (left)
25833	Volume of grey matter in Juxtapositional Lobule Cortex (formerly Supplementary Motor Cortex) (right)
25834	Volume of grey matter in Subcallosal Cortex (left)
25835	Volume of grey matter in Subcallosal Cortex (right)
25836	Volume of grey matter in Paracingulate Gyrus (left)
25837	Volume of grey matter in Paracingulate Gyrus (right)
25842	Volume of grey matter in Precuneous Cortex (left)
25843	Volume of grey matter in Precuneous Cortex (right)
25844	Volume of grey matter in Cuneal Cortex (left)
25845	Volume of grey matter in Cuneal Cortex (right)
25846	Volume of grey matter in Frontal Orbital Cortex (left)
25847	Volume of grey matter in Frontal Orbital Cortex (right)
25848	Volume of grey matter in Parahippocampal Gyrus, anterior division (left)
25849	Volume of grey matter in Parahippocampal Gyrus, anterior division (right)
25850	Volume of grey matter in Parahippocampal Gyrus, posterior division (left)
25851	Volume of grey matter in Parahippocampal Gyrus, posterior division (right)
25852	Volume of grey matter in Lingual Gyrus (left)
25853	Volume of grey matter in Lingual Gyrus (right)
25854	Volume of grey matter in Temporal Fusiform Cortex, anterior division (left)

25855 Volume of grey matter in Temporal Fusiform Cortex, anterior division (right)
25856 Volume of grey matter in Temporal Fusiform Cortex, posterior division (left)
25857 Volume of grey matter in Temporal Fusiform Cortex, posterior division (right)
25858 Volume of grey matter in Temporal Occipital Fusiform Cortex (left)
25859 Volume of grey matter in Temporal Occipital Fusiform Cortex (right)
25860 Volume of grey matter in Occipital Fusiform Gyrus (left)
25861 Volume of grey matter in Occipital Fusiform Gyrus (right)
25862 Volume of grey matter in Frontal Operculum Cortex (left)
25863 Volume of grey matter in Frontal Operculum Cortex (right)
25864 Volume of grey matter in Central Opercular Cortex (left)
25865 Volume of grey matter in Central Opercular Cortex (right)
25866 Volume of grey matter in Parietal Operculum Cortex (left)
25867 Volume of grey matter in Parietal Operculum Cortex (right)
25868 Volume of grey matter in Planum Polare (left)
25869 Volume of grey matter in Planum Polare (right)
25870 Volume of grey matter in Heschl's Gyrus (includes H1 and H2) (left)
25871 Volume of grey matter in Heschl's Gyrus (includes H1 and H2) (right)
25872 Volume of grey matter in Planum Temporale (left)
25873 Volume of grey matter in Planum Temporale (right)
25874 Volume of grey matter in Supracalcarine Cortex (left)
25875 Volume of grey matter in Supracalcarine Cortex (right)
25876 Volume of grey matter in Occipital Pole (left)
25877 Volume of grey matter in Occipital Pole (right)
25878 Volume of grey matter in Thalamus (left)
25879 Volume of grey matter in Thalamus (right)
25880 Volume of grey matter in Caudate (left)
25881 Volume of grey matter in Caudate (right)
25882 Volume of grey matter in Putamen (left)
25883 Volume of grey matter in Putamen (right)
25884 Volume of grey matter in Pallidum (left)
25885 Volume of grey matter in Pallidum (right)
25886 Volume of grey matter in Hippocampus (left)
25887 Volume of grey matter in Hippocampus (right)
25888 Volume of grey matter in Amygdala (left)
25889 Volume of grey matter in Amygdala (right)
25890 Volume of grey matter in Ventral Striatum (left)
25891 Volume of grey matter in Ventral Striatum (right)
25892 Volume of grey matter in Brain-Stem
25893 Volume of grey matter in I-IV Cerebellum (left)
25894 Volume of grey matter in I-IV Cerebellum (right)
25895 Volume of grey matter in V Cerebellum (left)
25896 Volume of grey matter in V Cerebellum (right)
25897 Volume of grey matter in VI Cerebellum (left)
25898 Volume of grey matter in VI Cerebellum (vermis)

25899	Volume of grey matter in VI Cerebellum (right)
25900	Volume of grey matter in Crus I Cerebellum (left)
25901	Volume of grey matter in Crus I Cerebellum (vermis)
25902	Volume of grey matter in Crus I Cerebellum (right)
25903	Volume of grey matter in Crus II Cerebellum (left)
25904	Volume of grey matter in Crus II Cerebellum (vermis)
25905	Volume of grey matter in Crus II Cerebellum (right)
25906	Volume of grey matter in VIIb Cerebellum (left)
25907	Volume of grey matter in VIIb Cerebellum (vermis)
25908	Volume of grey matter in VIIb Cerebellum (right)
25909	Volume of grey matter in VIIIa Cerebellum (left)
25910	Volume of grey matter in VIIIa Cerebellum (vermis)
25911	Volume of grey matter in VIIIa Cerebellum (right)
25912	Volume of grey matter in VIIIb Cerebellum (left)
25913	Volume of grey matter in VIIIb Cerebellum (vermis)
25914	Volume of grey matter in VIIIb Cerebellum (right)
25915	Volume of grey matter in IX Cerebellum (left)
25916	Volume of grey matter in IX Cerebellum (vermis)
25917	Volume of grey matter in IX Cerebellum (right)
25918	Volume of grey matter in X Cerebellum (left)
25919	Volume of grey matter in X Cerebellum (vermis)
25920	Volume of grey matter in X Cerebellum (right)

Supplementary Results

Table S2. Characteristics of UK Biobank Participants with or without depressive symptoms in cross-sectional study.

Traits	Participants free of depressive symptoms at baseline (N = 133726)	Participants with depressive symptoms at baseline (N = 11705)	*P*-values
**Age**	55.43(7.68)	53.69(7.36)	<0.001
**Sex**			<0.001
Female	73235(90.54%)	7653(9.46%)	
Male	60491(93.72%)	4052(6.28%)	
**BMI**	26.59(4.42)	27.70(5.42)	<0.001
**Average total household income**			<0.001
Less than 18,000	14499(85.89%)	2382(14.11%)	
18,000 to 30,999	29754(90.96%)	2956(9.04%)	
31,000 to 51,999	39792(92.28%)	3331(7.72%)	
52,000 to 100,000	37972(94.02%)	2417(5.98%)	
Greater than 100,000	11709(94.98%)	619(5.02%)	
**Smoke**			<0.001
Never	78821(92.56%)	6336(7.44%)	
Previous	45977(91.86%)	4073(8.14%)	
Current	8928(87.32%)	1296(12.68%)	
**Ethnic background**			<0.001
White	124062(92.05%)	10715(7.95%)	
Mixed	3869(91.57%)	356(8.43%)	
Asian or Asian British	5375(90.35%)	574(9.65%)	
Black or Black British	420(87.50%)	60(12.50%)	
Chinese			
Other ethnic group			
**Education**			<0.001
College or University degree	67631(92.52%)	5464(7.48%)	
A levels/AS levels or equivalent	19321(91.46%)	1805(8.54%)	
O levels/GCSEs or equivalent	27538(91.37%)	2600(8.63%)	
CSEs or equivalent	5089(89.36%)	606(10.64%)	
NVQ or HND or HNC or equivalent	7179(92.10%)	616(7.90%)	
Other professional qualifications eg: nursing, teaching	6968(91.90%)	614(8.10%)	

**Drink**			
Never	3166(89.66%)	365(10.34%)	
Previous	3016(82.99%)	618(17.01%)	
Current	127544(92.25%)	10722(7.75%)	
**Physical activity**			<0.001
Walking for pleasure (not as a means of transport)	74633(92.53%)	6025(7.47%)	
Other exercises (eg: swimming, cycling, keep fit, bowling)	11789(91.95%)	1032(8.05%)	
Strenuous sports	859(94.19%)	53(5.81%)	
Light DIY (eg: pruning, watering the lawn)	4905(88.11%)	662(11.89%)	
Heavy DIY (eg: weeding, lawn mowing, carpentry, digging)	1459(90.06%)	161(9.94%)	
**Systolic blood pressure**	136.50(18.09)	133.07(17.44)	<0.001
**Diastolic blood pressure**	81.75(9.94)	81.26(10.05)	<0.001
**Glucose**	3.48(0.99)	3.52(1.09)	0.056
**Total Triglycerides**	1.25(0.55)	1.32(0.60)	<0.001
**Total Cholesterol**	4.61(0.91)	4.62(0.90)	0.68
**HDL Cholesterol**	1.33(0.32)	1.32(0.32)	0.005

Mean (SD) was used in continuous variables; number (%) was used in categorized variables.

Table S3. Cox Regression Results for food preferences and depression (HR, 95%CI, and Significance) in female participants.

Variable	Model 1 HR (95%CI)	Model 2 HR (95%CI)	Model 3 HR (95%CI)
**Liking for bitter foods**			
**1**	1.305(1.169~1.457)**	1.258(1.126~1.405)**	1.136(0.873~1.478)
**2**	1.032(0.899~1.183)	1.018(0.887~1.167)	0.734(0.514~1.049)
**3**	0.981(0.857~1.123)	0.977(0.854~1.118)	0.926(0.670~1.281)
**4**	0.891(0.778~1.021)	0.894(0.781~1.024)	0.700(0.495~0.991)*
**5**	Ref	Ref	Ref
**6**	0.877(0.769~1.001)	0.893(0.782~1.019)	0.708(0.503~0.996)*
**7**	0.817(0.711~0.940)**	0.836(0.727~0.961)*	0.752(0.530~1.068)
**8**	0.770(0.631~0.939)**	0.787(0.646~0.960)*	0.761(0.463~1.248)
**9**	1.026(0.810~1.301)	1.047(0.826~1.328)	0.597(0.278~1.284)
**Liking for salty foods**			
**1**	1.049(0.908~1.211)	1.012(0.876~1.169)	0.851(0.606~1.195)
**2**	1.067(0.909~1.253)	1.044(0.889~1.226)	0.893(0.609~1.311)
**3**	1.040(0.889~1.216)	1.019(0.871~1.192)	1.026(0.721~1.459)

**4**	1.018(0.872~1.189)	1.020(0.874~1.191)	1.145(0.818~1.604)
**5**	Ref	Ref	Ref
**6**	0.890(0.771~1.027)	0.921(0.798~1.063)	0.820(0.584~1.152)
**7**	0.883(0.765~1.020)	0.921(0.798~1.064)	0.877(0.629~1.222)
**8**	0.880(0.742~1.043)	0.937(0.790~1.111)	0.672(0.440~1.024)
**9**	1.009(0.840~1.210)	1.084(0.903~1.301)	0.917(0.592~1.423)
**Liking for spicy foods**			
**1**	1.151(0.975~1.358)	1.134(0.961~1.338)	0.925(0.629~1.360)
**2**	1.242(1.029~1.498)*	1.229(1.018~1.483)*	0.823(0.513~1.321)
**3**	0.951(0.781~1.158)	0.948(0.779~1.154)	0.718(0.445~1.158)
**4**	0.927(0.767~1.120)	0.934(0.773~1.129)	0.745(0.473~1.173)
**5**	Ref	Ref	Ref
**6**	0.839(0.716~0.982)*	0.873(0.745~1.022)	0.737(0.508~1.069)
**7**	0.761(0.655~0.885)**	0.797(0.686~0.928)**	0.607(0.423~0.870)**
**8**	0.728(0.622~0.853)**	0.774(0.660~0.907)**	0.639(0.439~0.931)*
**9**	0.816(0.699~0.952)**	0.886(0.758~1.034)	0.719(0.500~1.033)
**Liking for sweet foods**			
**1**	0.962(0.778~1.189)	0.928(0.751~1.148)	0.648(0.349~1.205)
**2**	0.914(0.751~1.113)	0.899(0.739~1.095)	0.982(0.608~1.589)
**3**	0.828(0.693~0.989)*	0.822(0.688~0.982)*	0.792(0.503~1.247)
**4**	0.910(0.772~1.073)	0.917(0.778~1.080)	1.057(0.705~1.586)
**5**	Ref	Ref	Ref
**6**	0.812(0.711~0.926)**	0.839(0.735~0.958)**	0.963(0.691~1.340)
**7**	0.869(0.764~0.987)*	0.905(0.796~1.029)	1.018(0.738~1.405)
**8**	0.932(0.811~1.070)	0.980(0.854~1.126)	1.067(0.755~1.507)
**9**	1.043(0.911~1.195)	1.127(0.983~1.291)	1.163(0.826~1.637)

Model 1 is a crude model, and Model 2 was adjusted for age. We further adjusted ethnic background, BMI, smoking status, drinking status, education levels, average total household income before tax, MET minutes per week for activity, hypertension, diabetes, TG, total cholesterol, and HDL-C in Model 3. *, *P*-value <0.05, **, *P*-value <0.001

Table S4. Cox Regression Results for food preferences and depression (HR, 95%CI, and Significance) in male participants.

Variable	Model 1 HR (95%CI)	Model 2 HR (95%CI)	Model 3 HR (95%CI)
**Liking for bitter foods**			
**1**	1.383(1.191~1.606)**	1.284(1.106~1.492)**	1.282(0.914~1.798)
**2**	1.191(1.019~1.391)*	1.115(0.954~1.303)	0.988(0.684~1.427)
**3**	1.103(0.958~1.270)	1.068(0.928~1.231)	1.079(0.777~1.498)
**4**	0.958(0.832~1.103)	0.946(0.822~1.090)	0.967(0.695~1.345)
**5**	Ref	Ref	Ref

**6**	0.845(0.739~0.966)*	0.876(0.766~1.002)	0.905(0.665~1.232)
**7**	0.857(0.751~0.979)*	0.883(0.773~1.008)	0.665(0.474~0.934)*
**8**	0.738(0.610~0.893)**	0.759(0.627~0.918)**	1.005(0.678~1.491)
**9**	0.901(0.672~1.207)	0.936(0.698~1.254)	0.848(0.430~1.671)
**Liking for salty foods**			
**1**	1.132(0.965~1.329)	1.068(0.910~1.254)	1.192(0.844~1.683)
**2**	0.835(0.698~1.000)	0.783(0.654~0.937)**	0.886(0.596~1.317)
**3**	0.913(0.776~1.073)	0.883(0.751~1.038)	0.942(0.654~1.356)
**4**	0.800(0.678~0.945)**	0.791(0.670~0.934)**	0.963(0.677~1.370)
**5**	Ref	Ref	Ref
**6**	0.838(0.724~0.970)*	0.862(0.745~0.998)*	0.974(0.709~1.339)
**7**	0.876(0.759~1.011)	0.926(0.802~1.069)	0.999(0.728~1.371)
**8**	0.787(0.655~0.946)*	0.830(0.691~0.997)*	0.742(0.489~1.125)
**9**	0.911(0.704~1.179)	0.978(0.756~1.265)	0.886(0.501~1.567)
**Liking for spicy foods**			
**1**	1.331(1.082~1.638)**	1.291(1.049~1.589)*	1.021(0.658~1.586)
**2**	0.984(0.766~1.264)	0.951(0.740~1.221)	0.575(0.317~1.046)
**3**	1.076(0.851~1.360)	1.058(0.837~1.337)	0.723(0.415~1.261)
**4**	0.945(0.748~1.194)	0.929(0.735~1.174)	0.664(0.396~1.112)
**5**	Ref	Ref	Ref
**6**	0.884(0.735~1.064)	0.928(0.771~1.117)	0.766(0.522~1.126)
**7**	0.850(0.717~1.007)	0.917(0.774~1.086)	0.656(0.462~0.931)*
**8**	0.750(0.633~0.890)**	0.839(0.707~0.995)*	0.603(0.423~0.860)**
**9**	0.815(0.686~0.968)*	0.957(0.805~1.137)	0.593(0.412~0.855)**
**Liking for sweet foods**			
**1**	1.134(0.916~1.405)	1.069(0.863~1.324)	1.625(1.046~2.523)*
**2**	1.033(0.852~1.251)	0.998(0.824~1.209)	1.344(0.875~2.065)
**3**	0.816(0.679~0.980)*	0.802(0.667~0.963)*	1.247(0.835~1.863)
**4**	0.988(0.836~1.167)	0.986(0.834~1.166)	0.962(0.634~1.460)
**5**	Ref	Ref	Ref
**6**	0.906(0.788~1.042)	0.940(0.818~1.081)	1.064(0.753~1.503)
**7**	0.881(0.770~1.009)	0.912(0.797~1.044)	1.054(0.756~1.470)
**8**	0.942(0.814~1.091)	0.979(0.846~1.133)	1.130(0.788~1.621)
**9**	1.061(0.898~1.253)	1.135(0.961~1.341)	1.333(0.894~1.987)

Model 1 is a crude model, and Model 2 was adjusted for age and sex. We further adjusted ethnic background, BMI, smoking status, drinking status, education levels, average total household income before tax, MET minutes per week for activity, hypertension, diabetes, TG, total cholesterol, and HDL-C in Model 3. *, *P*-value <0.05, **, *P*-value <0.001

Table S5. Cox Regression Results for food preferences and depression (HR, 95%CI, and Significance) in <60 year-old participants.

Variable	Model 1 HR (95%CI)	Model 2 HR (95%CI)	Model 3 HR (95%CI)
**Liking for bitter foods**			
**1**	1.387(1.227~1.568)**	1.352(1.194~1.530)**	1.162(0.863~1.564)
**2**	1.045(0.901~1.212)	1.029(0.887~1.194)	0.594(0.387~0.913)*
**3**	1.087(0.950~1.243)	1.079(0.943~1.234)	0.945(0.678~1.317)
**4**	0.890(0.776~1.021)	0.886(0.772~1.016)	0.714(0.506~1.009)
**5**	Ref		Ref
**6**	0.908(0.801~1.030)	0.910(0.803~1.032)	0.769(0.562~1.052)
**7**	0.828(0.726~0.944)**	0.832(0.729~0.948)**	0.694(0.498~0.968)*
**8**	0.784(0.653~0.943)**	0.789(0.656~0.948)*	0.697(0.435~1.118)
**9**	1.043(0.821~1.325)	1.035(0.815~1.316)	0.566(0.264~1.212)
**Liking for salty foods**			
**1**	1.160(0.995~1.353)	1.136(0.974~1.325)	1.232(0.873~1.737)
**2**	0.968(0.812~1.153)	0.958(0.804~1.142)	0.664(0.417~1.056)
**3**	0.999(0.849~1.176)	0.997(0.847~1.173)	0.855(0.578~1.264)
**4**	0.943(0.803~1.107)	0.939(0.800~1.103)	0.975(0.678~1.401)
**5**	Ref	Ref	Ref
**6**	0.955(0.829~1.099)	0.950(0.825~1.094)	0.813(0.580~1.139)
**7**	0.928(0.806~1.068)	0.925(0.804~1.065)	0.905(0.655~1.252)
**8**	0.878(0.741~1.039)	0.869(0.734~1.029)	0.706(0.468~1.063)
**9**	1.124(0.933~1.355)	1.086(0.901~1.310)	1.065(0.692~1.639)
**Liking for spicy foods**			
**1**	1.189(0.980~1.444)	1.173(0.966~1.424)	0.800(0.500~1.281)
**2**	1.128(0.899~1.414)	1.119(0.892~1.403)	0.659(0.363~1.197)
**3**	1.117(0.898~1.388)	1.110(0.893~1.380)	0.905(0.540~1.519)
**4**	1.008(0.815~1.247)	0.999(0.808~1.236)	0.610(0.355~1.048)
**5**	Ref	Ref	Ref
**6**	0.851(0.715~1.013)	0.848(0.712~1.009)	0.748(0.500~1.118)
**7**	0.795(0.675~0.936)**	0.799(0.678~0.941)**	0.601(0.410~0.883)**
**8**	0.711(0.602~0.841)**	0.723(0.612~0.855)**	0.575(0.390~0.848)**
**9**	0.887(0.754~1.043)	0.897(0.762~1.055)	0.665(0.458~0.967)*
**Liking for sweet foods**			
**1**	0.936(0.740~1.186)	0.932(0.736~1.180)	0.842(0.460~1.540)
**2**	0.939(0.766~1.150)	0.940(0.767~1.152)	1.054(0.644~1.726)
**3**	0.853(0.710~1.023)	0.853(0.711~1.023)	1.052(0.682~1.622)

**4**	0.868(0.731~1.030)	0.865(0.729~1.027)	0.808(0.516~1.263)
**5**	Ref	Ref	Ref
**6**	0.836(0.730~0.957)**	0.829(0.724~0.949)**	0.892(0.632~1.259)
**7**	0.866(0.760~0.987)*	0.860(0.755~0.981)*	0.935(0.671~1.303)
**8**	0.980(0.853~1.127)	0.974(0.848~1.119)	1.037(0.730~1.474)
**9**	1.157(1.007~1.330)*	1.128(0.981~1.297)	1.048(0.730~1.505)

Model 1 is crude model, and Model 2 adjusted with sex. We further adjusted ethnic background, BMI, smoking status, drinking status, education levels, average total household income before tax, MET minutes per week for activity, hypertension, diabetes, TG, total cholesterol, and HDL-C in Model 3. *, *P*-value <0.05,**, *P*-value <0.001

Table S6. Cox Regression Results for food preferences and depression (HR, 95%CI, and Significance) in ≥60 year-old participants.

Variable	Model 1 HR (95%CI)	Model 2 HR (95%CI)	Model 3 HR (95%CI)
**Liking for bitter foods**			
**1**	1.229(1.093~1.382)**	1.271(1.128~1.431)**	1.233(0.931~1.634)
**2**	1.073(0.934~1.232)	1.086(0.946~1.248)	1.077(0.783~1.482)
**3**	1.000(0.873~1.144)	1.005(0.878~1.150)	1.132(0.834~1.537)
**4**	0.954(0.834~1.091)	0.956(0.836~1.094)	0.951(0.690~1.310)
**5**	Ref	Ref	Ref
**6**	0.853(0.744~0.978)*	0.852(0.743~0.977)*	0.870(0.633~1.197)
**7**	0.884(0.772~1.013)	0.878(0.766~1.006)	0.712(0.503~1.007)
**8**	0.782(0.643~0.950)*	0.776(0.639~0.944)*	1.134(0.768~1.673)
**9**	0.960(0.731~1.261)	0.972(0.740~1.276)	1.073(0.578~1.991)
**Liking for salty foods**			
**1**	0.982(0.853~1.131)	0.995(0.864~1.145)	0.858(0.616~1.194)
**2**	0.901(0.770~1.055)	0.904(0.772~1.058)	1.048(0.744~1.475)
**3**	0.914(0.787~1.061)	0.915(0.788~1.063)	1.107(0.801~1.531)
**4**	0.870(0.746~1.014)	0.871(0.747~1.015)	1.073(0.778~1.481)
**5**	Ref	Ref	Ref
**6**	0.820(0.710~0.947)**	0.820(0.710~0.947)**	0.997(0.733~1.357)
**7**	0.913(0.793~1.051)	0.913(0.793~1.051)	0.974(0.713~1.329)
**8**	0.896(0.750~1.071)	0.896(0.750~1.071)	0.753(0.501~1.133)
**9**	0.919(0.730~1.158)	0.932(0.740~1.174)	0.624(0.347~1.121)
**Liking for spicy foods**			
**1**	1.208(1.023~1.426)*	1.226(1.038~1.448)*	1.145(0.798~1.643)
**2**	1.148(0.948~1.390)	1.155(0.954~1.398)	0.803(0.507~1.272)
**3**	0.903(0.738~1.104)	0.908(0.742~1.110)	0.609(0.368~1.006)

**4**	0.865(0.710~1.053)	0.869(0.714~1.058)	0.816(0.532~1.252)
**5**	Ref	Ref	Ref
**6**	0.913(0.778~1.072)	0.913(0.777~1.071)	0.785(0.551~1.119)
**7**	0.865(0.744~1.005)	0.858(0.738~0.997)*	0.706(0.508~0.980)*
**8**	0.906(0.778~1.055)	0.891(0.764~1.037)	0.726(0.519~1.016)
**9**	0.851(0.725~0.999)*	0.841(0.717~0.988)*	0.625(0.437~0.895)*
**Liking for sweet foods**			
**1**	1.041(0.862~1.256)	1.043(0.864~1.260)	1.292(0.847~1.971)
**2**	0.928(0.775~1.112)	0.928(0.775~1.111)	1.165(0.774~1.753)
**3**	0.748(0.629~0.889)**	0.748(0.630~0.890)**	0.926(0.623~1.379)
**4**	0.990(0.848~1.155)	0.990(0.848~1.155)	1.045(0.717~1.523)
**5**	Ref	Ref	Ref
**6**	0.900(0.790~1.026)	0.904(0.794~1.030)	1.041(0.757~1.431)
**7**	0.919(0.810~1.043)	0.920(0.811~1.044)	1.082(0.797~1.471)
**8**	0.966(0.841~1.110)	0.966(0.841~1.110)	1.079(0.768~1.515)
**9**	1.049(0.901~1.221)	1.061(0.911~1.236)	1.389(0.978~1.971)

Model 1 is a crude model, and Model 2 was adjusted for sex. We further adjusted ethnic background, BMI, smoking status, drinking status, education levels, average total household income before tax, MET minutes per week for activity, hypertension, diabetes, TG, total cholesterol, and HDL-C in Model 3. *, *P*-value <0.05, **, *P*-value <0.001

Table S7. Cox Regression Results for food preferences and depression (HR, 95%CI, and Significance) in participants with normal BMI.

**Variable**	**Model 1 HR (95%CI)**	**Model 2 HR (95%CI)**	**Model 3 HR (95%CI)**
**Liking for bitter foods**			
**1**	1.258(1.070~1.479)**	1.192(1.013~1.404)*	1.074(0.713~1.617)
**2**	0.991(0.819~1.200)	0.961(0.794~1.164)	0.746(0.447~1.243)
**3**	0.916(0.760~1.104)	0.901(0.748~1.086)	1.022(0.656~1.591)
**4**	0.871(0.726~1.043)	0.871(0.727~1.044)	0.637(0.386~1.051)
**5**	Ref	Ref	Ref
**6**	0.898(0.759~1.063)	0.918(0.775~1.087)	0.875(0.568~1.347)
**7**	0.865(0.729~1.026)	0.896(0.755~1.063)	0.703(0.439~1.124)
**8**	0.704(0.549~0.904)**	0.729(0.568~0.935)*	0.640(0.329~1.247)
**9**	0.912(0.651~1.278)	0.937(0.669~1.313)	0.317(0.077~1.298)
**Liking for salty foods**			
**1**	1.130(0.927~1.377)	1.065(0.874~1.299)	0.883(0.551~1.416)
**2**	1.060(0.855~1.314)	1.008(0.813~1.249)	0.831(0.495~1.395)
**3**	1.094(0.890~1.344)	1.057(0.860~1.299)	0.823(0.494~1.371)

**4**	1.030(0.838~1.266)	1.026(0.834~1.261)	1.112(0.695~1.780)
**5**	Ref	Ref	Ref
**6**	0.930(0.768~1.128)	0.974(0.804~1.181)	0.777(0.485~1.244)
**7**	0.888(0.731~1.078)	0.950(0.782~1.154)	1.012(0.652~1.571)
**8**	0.768(0.599~0.985)*	0.845(0.659~1.084)	0.670(0.362~1.238)
**9**	0.965(0.729~1.279)	1.086(0.819~1.441)	0.320(0.114~0.902)*
**Liking for spicy foods**			
**1**	1.242(0.981~1.571)	1.217(0.962~1.540)	1.515(0.836~2.747)
**2**	1.143(0.876~1.492)	1.120(0.858~1.462)	1.059(0.521~2.155)
**3**	1.047(0.801~1.367)	1.049(0.803~1.370)	1.074(0.537~2.151)
**4**	0.953(0.731~1.243)	0.963(0.739~1.256)	0.661(0.298~1.466)
**5**	Ref	Ref	Ref
**6**	0.855(0.688~1.064)	0.913(0.734~1.136)	1.263(0.728~2.191)
**7**	0.775(0.631~0.953)*	0.835(0.679~1.026)	0.820(0.476~1.412)
**8**	0.734(0.594~0.907)**	0.810(0.654~1.001)	0.820(0.468~1.438)
**9**	0.778(0.628~0.963)*	0.897(0.723~1.112)	0.879(0.503~1.538)
**Liking for sweet foods**			
**1**	1.180(0.913~1.526)	1.112(0.860~1.438)	1.099(0.553~2.185)
**2**	0.989(0.776~1.261)	0.957(0.751~1.219)	1.098(0.601~2.003)
**3**	0.791(0.628~0.996)*	0.779(0.618~0.981)*	0.768(0.416~1.420)
**4**	0.830(0.665~1.035)	0.832(0.667~1.038)	0.538(0.271~1.067)
**5**	Ref	Ref	Ref
**6**	0.838(0.704~0.998)*	0.879(0.738~1.047)	0.853(0.534~1.362)
**7**	0.866(0.730~1.026)	0.912(0.769~1.081)	1.013(0.651~1.578)
**8**	0.915(0.759~1.102)	0.967(0.802~1.165)	1.272(0.801~2.017)
**9**	0.908(0.742~1.111)	1.010(0.825~1.236)	1.040(0.614~1.762)

Model 1 is a crude model, and Model 2 was adjusted for age and sex. We further adjusted ethnic background, smoking status, drinking status, education levels, average total household income before tax, MET minutes per week for activity, hypertension, diabetes, TG, total cholesterol, and HDL-C in Model 3. *, *P*-value <0.05,**, *P*-value <0.001

Table S8. Cox Regression Results for food preferences and depression (HR, 95%CI, and Significance) in overweight participants.

Variable	Model 1 HR (95%CI)	Model 2 HR (95%CI)	Model 3 HR (95%CI)
**Liking for bitter foods**			
**1**	1.241(1.079~1.426)**	1.166(1.012~1.342)*	1.251(0.916~1.709)
**2**	1.179(1.008~1.378)*	1.134(0.970~1.327)	0.955(0.659~1.385)
**3**	1.032(0.887~1.201)	1.016(0.873~1.182)	0.833(0.573~1.211)

**4**	0.944(0.812~1.098)	0.941(0.809~1.094)	0.836(0.583~1.198)
**5**	Ref	Ref	Ref
**6**	0.834(0.720~0.967)*	0.860(0.742~0.996)*	0.769(0.543~1.089)
**7**	0.703(0.600~0.824)**	0.720(0.615~0.844)**	0.552(0.371~0.819)**
**8**	0.792(0.641~0.978)*	0.808(0.654~0.997)*	0.889(0.561~1.409)
**9**	0.851(0.622~1.164)	0.873(0.638~1.194)	0.541(0.220~1.329)
**Liking for salty foods**			
**1**	1.079(0.916~1.271)	1.019(0.864~1.201)	1.091(0.763~1.559)
**2**	0.920(0.763~1.110)	0.885(0.734~1.067)	0.779(0.507~1.198)
**3**	0.833(0.697~0.997)*	0.812(0.679~0.971)*	0.759(0.505~1.141)
**4**	0.851(0.714~1.015)	0.848(0.712~1.011)	1.075(0.748~1.545)
**5**	Ref	Ref	Ref
**6**	0.820(0.701~0.961)*	0.846(0.722~0.991)*	0.920(0.649~1.304)
**7**	0.826(0.706~0.967)*	0.861(0.736~1.008)	0.854(0.601~1.215)
**8**	0.783(0.645~0.950)*	0.825(0.679~1.001)	0.715(0.451~1.131)
**9**	0.868(0.683~1.102)	0.925(0.727~1.176)	1.023(0.613~1.708)
**Liking for spicy foods**			
**1**	1.211(0.987~1.486)	1.179(0.961~1.447)	0.881(0.568~1.367)
**2**	1.101(0.865~1.402)	1.077(0.846~1.371)	0.568(0.316~1.020)
**3**	0.932(0.730~1.191)	0.922(0.722~1.177)	0.554(0.308~0.995)*
**4**	0.832(0.654~1.057)	0.827(0.651~1.052)	0.804(0.495~1.306)
**5**	Ref	Ref	Ref
**6**	0.868(0.719~1.049)	0.909(0.752~1.098)	0.688(0.462~1.026)
**7**	0.800(0.670~0.954)*	0.851(0.713~1.017)	0.528(0.361~0.774)**
**8**	0.705(0.588~0.845)**	0.774(0.645~0.928)**	0.589(0.404~0.858)**
**9**	0.823(0.688~0.984)*	0.932(0.779~1.116)	0.615(0.420~0.901)*
**Liking for sweet foods**			
**1**	0.913(0.712~1.172)	0.879(0.685~1.128)	1.093(0.646~1.847)
**2**	0.951(0.764~1.183)	0.935(0.751~1.164)	1.116(0.694~1.794)
**3**	0.814(0.665~0.996)*	0.805(0.657~0.985)*	1.043(0.671~1.622)
**4**	0.993(0.828~1.190)	0.998(0.833~1.196)	0.973(0.627~1.513)
**5**	Ref	Ref	Ref
**6**	0.852(0.733~0.991)*	0.878(0.755~1.020)	0.883(0.612~1.273)
**7**	0.883(0.764~1.021)	0.914(0.791~1.057)	1.048(0.743~1.478)
**8**	0.880(0.751~1.031)	0.920(0.785~1.078)	0.881(0.595~1.306)
**9**	1.071(0.910~1.260)	1.145(0.972~1.348)	1.215(0.827~1.787)

Model 1 is a crude model, and Model 2 was adjusted for age and sex. We further adjusted ethnic background, smoking status, drinking status, education levels, average total household income before tax, MET minutes per week for activity, hypertension, diabetes, TG, total cholesterol, and HDL-C in Model 3. *, *P*-value <0.05,**, *P*-value <0.001.

Table S9. Cox Regression Results for food preferences and depression (HR, 95%CI, and Significance) in obesity participants.

Variable	Model 1 HR (95%CI)	Model 2 HR (95%CI)	Model 3 HR (95%CI)
**Liking for bitter foods**			
**1**	1.430(1.218~1.679)**	1.382(1.174~1.626)**	1.227(0.831~1.813)
**2**	1.143(0.934~1.399)	1.119(0.914~1.370)	0.851(0.516~1.405)
**3**	1.196(0.997~1.434)	1.187(0.990~1.424)	1.274(0.849~1.911)
**4**	0.952(0.786~1.154)	0.951(0.785~1.152)	0.971(0.625~1.509)
**5**	Ref	Ref	Ref
**6**	0.889(0.739~1.070)	0.911(0.757~1.096)	0.775(0.496~1.212)
**7**	1.092(0.913~1.306)	1.104(0.923~1.321)	1.033(0.675~1.582)
**8**	0.887(0.679~1.161)	0.899(0.688~1.176)	1.145(0.663~1.976)
**9**	1.276(0.930~1.751)	1.315(0.958~1.806)	1.603(0.797~3.224)
**Liking for salty foods**			
**1**	1.123(0.914~1.380)	1.078(0.877~1.326)	1.004(0.623~1.617)
**2**	1.033(0.815~1.311)	1.002(0.790~1.271)	1.113(0.654~1.896)
**3**	1.211(0.980~1.498)	1.185(0.958~1.465)	0.725(0.562~0.936)*
**4**	0.942(0.754~1.177)	0.937(0.750~1.171)	0.971(0.601~1.569)
**5**	Ref	Ref	Ref
**6**	0.924(0.759~1.124)	0.939(0.771~1.142)	1.026(0.666~1.581)
**7**	0.949(0.784~1.150)	0.976(0.806~1.183)	1.007(0.650~1.561)
**8**	0.967(0.771~1.212)	0.998(0.795~1.251)	0.846(0.506~1.415)
**9**	1.092(0.847~1.409)	1.122(0.869~1.447)	1.292(0.738~2.262)
**Liking for spicy foods**			
**1**	1.152(0.903~1.471)	1.128(0.883~1.440)	0.734(0.433~1.241)
**2**	1.284(0.964~1.711)	1.255(0.942~1.672)	0.557(0.272~1.144)
**3**	1.060(0.794~1.415)	1.029(0.771~1.374)	0.692(0.359~1.331)
**4**	1.060(0.806~1.396)	1.061(0.806~1.397)	0.597(0.324~1.100)
**5**	Ref	Ref	Ref
**6**	0.895(0.710~1.128)	0.917(0.728~1.155)	0.725(0.562~0.936)*
**7**	0.883(0.713~1.094)	0.917(0.739~1.136)	0.657(0.424~1.018)
**8**	0.828(0.667~1.029)	0.868(0.698~1.079)	0.725(0.562~0.936)*
**9**	0.811(0.654~1.007)	0.873(0.703~1.085)	0.545(0.343~0.865)**
**Liking for sweet foods**			
**1**	1.162(0.867~1.559)	1.120(0.835~1.502)	1.377(0.714~2.654)
**2**	1.118(0.856~1.461)	1.095(0.838~1.431)	1.174(0.609~2.262)
**3**	0.961(0.752~1.228)	0.952(0.745~1.216)	1.290(0.741~2.248)

**4**	1.087(0.871~1.358)	1.086(0.869~1.356)	1.427(0.853~2.388)
**5**	Ref	Ref	Ref
**6**	0.936(0.778~1.127)	0.953(0.791~1.147)	1.389(0.884~2.182)
**7**	0.916(0.766~1.095)	0.936(0.783~1.120)	1.054(0.663~1.675)
**8**	1.055(0.872~1.275)	1.091(0.902~1.320)	1.345(0.836~2.163)
**9**	1.099(0.908~1.330)	1.153(0.951~1.399)	1.476(0.909~2.397)

Model 1 is a crude model, and Model 2 was adjusted for age and sex. We further adjusted ethnic background, smoking status, drinking status, education levels, average total household income before tax, MET minutes per week for activity, hypertension, diabetes, TG, total cholesterol, and HDL-C in Model 3. *, *P*-value <0.05,**, *P*-value <0.001.

Table S10. Sensitive analysis of cox regression results for food preferences and depression (HR, 95%CI, and Significance) without <2-year follow-up.

Variable	Model 1 HR (95%CI)	Model 2 HR (95%CI)	Model 3 HR (95%CI)
**Liking for bitter foods**			
**1**	1.323(1.212~1.445)**	1.272(1.164~1.391)**	1.179(0.957~1.454)
**2**	1.099(0.990~1.219)	1.072(0.966~1.190)	0.851(0.658~1.102)
**3**	1.027(0.930~1.134)	1.016(0.920~1.122)	0.967(0.765~1.222)
**4**	0.914(0.827~1.010)	0.914(0.828~1.010)	0.787(0.617~1.005)
**5**	Ref	Ref	Ref
**6**	0.854(0.776~0.940)**	0.876(0.796~0.964)**	0.795(0.631~1.000)
**7**	0.837(0.759~0.922)**	0.858(0.778~0.946)**	0.667(0.520~0.856)**
**8**	0.752(0.654~0.865)**	0.770(0.669~0.885)**	0.878(0.644~1.197)
**9**	0.993(0.825~1.194)	1.027(0.853~1.235)	0.748(0.451~1.242)
**Liking for salty foods**			
**1**	1.074(0.964~1.197)	1.024(0.919~1.141)	1.005(0.787~1.284)
**2**	0.955(0.846~1.078)	0.918(0.813~1.036)	0.906(0.687~1.196)
**3**	0.978(0.873~1.096)	0.952(0.849~1.067)	0.990(0.767~1.279)
**4**	0.911(0.812~1.021)	0.908(0.810~1.019)	1.065(0.833~1.360)
**5**	Ref	Ref	Ref
**6**	0.852(0.768~0.946)**	0.883(0.796~0.980)*	0.873(0.689~1.106)
**7**	0.878(0.792~0.973)*	0.924(0.834~1.024)	0.926(0.734~1.168)
**8**	0.836(0.737~0.949)**	0.894(0.788~1.014)	0.704(0.521~0.952)*
**9**	0.963(0.830~1.117)	1.051(0.905~1.219)	0.902(0.635~1.282)
**Liking for spicy foods**			
**1**	1.212(1.063~1.381)**	1.192(1.045~1.359)**	1.003(0.748~1.345)
**2**	1.140(0.980~1.328)	1.121(0.963~1.306)	0.717(0.492~1.044)
**3**	0.993(0.852~1.158)	0.987(0.847~1.151)	0.717(0.495~1.039)
**4**	0.944(0.813~1.095)	0.948(0.817~1.101)	0.715(0.506~1.012)

**5**	Ref	Ref	Ref
**6**	0.860(0.762~0.972)*	0.905(0.801~1.023)	0.771(0.586~1.012)
**7**	0.800(0.714~0.897)**	0.850(0.758~0.953)**	0.662(0.512~0.855)**
**8**	0.742(0.660~0.834)**	0.803(0.714~0.903)**	0.644(0.496~0.837)**
**9**	0.819(0.729~0.920)**	0.920(0.819~1.034)**	0.662(0.508~0.861)**
**Liking for sweet foods**			
**1**	1.039(0.891~1.210)	0.990(0.850~1.154)	1.182(0.831~1.680)
**2**	0.970(0.844~1.115)	0.946(0.823~1.088)	1.154(0.835~1.596)
**3**	0.817(0.718~0.930)**	0.807(0.709~0.919)**	1.009(0.745~1.366)
**4**	0.965(0.857~1.086)	0.969(0.861~1.091)	0.992(0.739~1.332)
**5**	Ref	Ref	Ref
**6**	0.847(0.769~0.934)**	0.881(0.799~0.972)**	0.997(0.782~1.272)
**7**	0.878(0.799~0.964)**	0.916(0.834~1.007)	1.037(0.820~1.311)
**8**	0.940(0.849~1.041)	0.990(0.894~1.097)	1.143(0.889~1.469)
**9**	1.049(0.944~1.165)	1.148(1.032~1.276)	1.252(0.964~1.628)

Model 1 is a crude model, and Model 2 was adjusted for age and sex. We further adjusted ethnic background, BMI, smoking status, drinking status, education levels, average total household income before tax, MET minutes per week for activity, hypertension, diabetes, TG, total cholesterol, and HDL-C in Model 3. *, *P*-value <0.05,**, *P*-value <0.001

Table S11. Sensitive analysis of cox regression results for food preferences and depression (HR, 95%CI, and Significance) with excluding extreme glucose levels.

Variable	Model 1 HR (95%CI)	Model 2 HR (95%CI)	Model 3 HR (95%CI)
**Liking for bitter foods**			
**1**	1.287(1.074~1.542)**	1.198(0.998~1.439)	1.167(0.936~1.454)
**2**	1.003(0.809~1.244)	0.961(0.775~1.192)	0.915(0.704~1.190)
**3**	0.972(0.791~1.195)	0.952(0.774~1.170)	0.936(0.728~1.202)
**4**	0.816(0.662~1.006)	0.818(0.664~1.009)	0.774(0.598~1.002)
**5**	Ref	Ref	Ref
**6**	0.736(0.600~0.904)**	0.753(0.614~0.925)**	0.771(0.603~0.986)*
**7**	0.680(0.550~0.841)**	0.704(0.569~0.870)**	0.657(0.503~0.858)**
**8**	0.768(0.579~1.018)	0.789(0.595~1.046)	0.912(0.657~1.266)
**9**	0.594(0.364~0.967)*	0.616(0.378~1.004)	0.603(0.329~1.105)
**Liking for salty foods**			
**1**	1.143(0.911~1.433)	1.068(0.851~1.341)	0.983(0.758~1.274)
**2**	0.971(0.754~1.250)	0.924(0.717~1.190)	0.879(0.656~1.177)
**3**	0.916(0.715~1.173)	0.896(0.700~1.148)	0.929(0.707~1.222)
**4**	0.951(0.750~1.206)	0.946(0.746~1.199)	1.025(0.789~1.330)

**5**	Ref	Ref	Ref
**6**	0.853(0.683~1.064)	0.885(0.709~1.104)	0.849(0.661~1.090)
**7**	0.874(0.702~1.088)	0.924(0.742~1.151)	0.927(0.726~1.183)
**8**	0.670(0.504~0.892)**	0.713(0.536~0.950)*	0.699(0.508~0.963)*
**9**	0.946(0.689~1.299)	1.009(0.734~1.387)	0.858(0.593~1.243)
**Liking for spicy foods**			
**1**	1.029(0.795~1.334)	0.993(0.766~1.287)	0.911(0.671~1.238)
**2**	0.890(0.653~1.213)	0.876(0.643~1.193)	0.714(0.488~1.046)
**3**	0.843(0.622~1.143)	0.840(0.620~1.140)	0.782(0.544~1.125)
**4**	0.626(0.455~0.860)**	0.634(0.461~0.871)**	0.654(0.456~0.939)*
**5**	Ref	Ref	Ref
**6**	0.715(0.563~0.908)**	0.755(0.594~0.959)*	0.710(0.536~0.941)*
**7**	0.584(0.466~0.733)**	0.626(0.499~0.786)**	0.564(0.432~0.738)**
**8**	0.566(0.449~0.714)**	0.625(0.496~0.789)**	0.553(0.420~0.729)**
**9**	0.662(0.528~0.831)**	0.760(0.604~0.955)*	0.631(0.481~0.828)**
**Liking for sweet foods**			
**1**	1.003(0.716~1.404)	0.959(0.685~1.343)	1.058(0.710~1.577)
**2**	0.987(0.735~1.325)	0.966(0.719~1.297)	1.058(0.745~1.503)
**3**	0.765(0.575~1.017)	0.755(0.567~1.004)	0.882(0.634~1.227)
**4**	0.884(0.683~1.144)	0.894(0.691~1.158)	0.947(0.693~1.293)
**5**	Ref	Ref	Ref
**6**	0.860(0.699~1.059)	0.894(0.726~1.101)	0.959(0.746~1.233)
**7**	0.915(0.749~1.118)	0.956(0.782~1.168)	0.962(0.754~1.229)
**8**	1.031(0.834~1.274)	1.091(0.883~1.349)	1.083(0.835~1.406)
**9**	1.150(0.922~1.433)	1.246(0.999~1.555)	1.230(0.940~1.610)

Model 1 is a crude model, and Model 2 was adjusted for age and sex. We further adjusted ethnic background, BMI, smoking status, drinking status, education levels, average total household income before tax, MET minutes per week for activity, hypertension, diabetes, TG, total cholesterol, and HDL-C in Model 3. *, *P*-value <0.05,**, *P*-value <0.001

Table S12. Sensitive analysis of cox regression results for food preferences and depression (HR, 95%CI, and Significance) with excluding extreme TG levels.

**Variable**	**Model 1 HR (95%CI)**	**Model 2 HR (95%CI)**	**Model 3 HR (95%CI)**
**Liking for bitter foods**			
**1**	1.348(1.127~1.612)**	1.250(1.042~1.498)*	1.200(0.964~1.493)
**2**	1.005(0.810~1.247)	0.964(0.777~1.197)	0.914(0.703~1.190)
**3**	0.980(0.796~1.207)	0.957(0.777~1.178)	0.918(0.712~1.183)
**4**	0.867(0.706~1.066)	0.867(0.706~1.066)	0.826(0.641~1.065)
**5**	Ref	Ref	Ref

**6**	0.792(0.647~0.969)*	0.810(0.662~0.990)*	0.813(0.638~1.037)
**7**	0.751(0.611~0.923)**	0.775(0.630~0.953)*	0.735(0.568~0.951)*
**8**	0.786(0.594~1.041)	0.809(0.611~1.071)	0.923(0.664~1.281)
**9**	0.676(0.426~1.075)	0.696(0.438~1.107)	0.729(0.416~1.277)
**Liking for salty foods**			
**1**	1.098(0.879~1.371)	1.026(0.821~1.282)	0.908(0.702~1.175)
**2**	0.931(0.726~1.195)	0.887(0.691~1.139)	0.842(0.631~1.125)
**3**	0.872(0.683~1.112)	0.852(0.668~1.087)	0.874(0.666~1.147)
**4**	0.898(0.710~1.135)	0.888(0.703~1.123)	0.957(0.739~1.240)
**5**	Ref	Ref	Ref
**6**	0.850(0.685~1.054)	0.877(0.707~1.088)	0.866(0.680~1.103)
**7**	0.867(0.701~1.073)	0.914(0.738~1.131)	0.914(0.720~1.161)
**8**	0.624(0.470~0.830)**	0.662(0.497~0.880)**	0.656(0.477~0.903)**
**9**	0.839(0.608~1.158)	0.889(0.644~1.229)	0.791(0.545~1.148)
**Liking for spicy foods**			
**1**	1.039(0.807~1.339)	0.997(0.774~1.285)	0.958(0.709~1.297)
**2**	0.860(0.634~1.167)	0.844(0.622~1.145)	0.751(0.516~1.092)
**3**	0.761(0.559~1.036)	0.754(0.553~1.026)	0.731(0.504~1.061)
**4**	0.620(0.454~0.847)**	0.627(0.459~0.856)**	0.669(0.468~0.958)*
**5**	Ref	Ref	Ref
**6**	0.701(0.554~0.886)**	0.738(0.583~0.933)*	0.732(0.554~0.968)*
**7**	0.579(0.463~0.723)**	0.617(0.493~0.771)**	0.595(0.456~0.777)**
**8**	0.569(0.453~0.713)**	0.625(0.497~0.784)**	0.576(0.438~0.757)**
**9**	0.632(0.505~0.792)**	0.718(0.573~0.900)**	0.619(0.471~0.813)**
**Liking for sweet foods**			
**1**	0.969(0.690~1.362)	0.929(0.661~1.306)	0.992(0.662~1.486)
**2**	1.029(0.769~1.378)	1.007(0.752~1.348)	1.122(0.795~1.585)
**3**	0.820(0.621~1.084)	0.810(0.613~1.070)	0.933(0.675~1.290)
**4**	0.945(0.733~1.219)	0.955(0.741~1.232)	1.006(0.740~1.368)
**5**	Ref	Ref	Ref
**6**	0.864(0.702~1.065)	0.896(0.727~1.103)	0.947(0.736~1.219)
**7**	0.965(0.791~1.178)	1.007(0.825~1.229)	1.018(0.799~1.297)
**8**	1.060(0.859~1.310)	1.120(0.906~1.383)	1.129(0.872~1.462)
**9**	1.177(0.945~1.465)	1.269(1.017~1.582)*	1.182(0.902~1.550)

Model 1 is a crude model, and Model 2 was adjusted for age and sex. We further adjusted ethnic background, BMI, smoking status, drinking status, education levels, average total household income before tax, MET minutes per week for activity, hypertension, diabetes, TG, total cholesterol, and HDL-C in Model 3. *, *P*-value <0.05,**, *P*-value <0.001

Table S13. Sensitive analysis of cox regression results for food preferences and depression (HR, 95%CI, and Significance) with excluding extreme blood pressures levels.

Variable	Model 1 HR (95%CI)	Model 2 HR (95%CI)	Model 3 HR (95%CI)
**Liking for bitter foods**			
**1**	1.319(1.203~1.447)**	1.255(1.143~1.379)**	1.162(1.037~1.304)*
**2**	1.118(1.003~1.246)*	1.083(0.972~1.207)	1.059(0.929~1.208)
**3**	1.038(0.936~1.151)	1.022(0.922~1.134)	1.032(0.911~1.170)
**4**	0.934(0.842~1.036)	0.933(0.841~1.035)	0.947(0.836~1.073)
**5**	Ref	Ref	Ref
**6**	0.865(0.783~0.956)**	0.886(0.802~0.979)*	0.881(0.780~0.995)*
**7**	0.841(0.759~0.931)**	0.861(0.778~0.954)**	0.862(0.761~0.977)*
**8**	0.779(0.675~0.900)**	0.796(0.690~0.919)**	0.878(0.742~1.040)
**9**	0.933(0.764~1.139)	0.959(0.785~1.171)	0.862(0.669~1.110)
**Liking for salty foods**			
**1**	1.086(0.970~1.215)	1.028(0.919~1.151)	1.001(0.880~1.139)
**2**	0.959(0.845~1.088)	0.919(0.810~1.043)	0.908(0.785~1.051)
**3**	0.970(0.861~1.093)	0.943(0.837~1.062)	0.981(0.857~1.123)
**4**	0.885(0.785~0.999)*	0.881(0.781~0.994)*	0.941(0.822~1.077)
**5**	Ref	Ref	Ref
**6**	0.877(0.787~0.977)*	0.907(0.814~1.010)	0.903(0.799~1.021)
**7**	0.890(0.799~0.990)*	0.930(0.835~1.035)	0.931(0.824~1.051)
**8**	0.805(0.704~0.920)**	0.853(0.746~0.975)*	0.862(0.742~1.002)
**9**	0.940(0.804~1.100)	1.009(0.862~1.182)	0.917(0.764~1.100)
**Liking for spicy foods**			
**1**	1.230(1.072~1.411)**	1.205(1.050~1.382)**	1.078(0.914~1.271)
**2**	1.135(0.967~1.331)	1.115(0.951~1.309)	1.050(0.866~1.272)
**3**	1.013(0.862~1.189)	1.003(0.854~1.177)	0.902(0.742~1.097)
**4**	0.925(0.791~1.083)	0.927(0.792~1.084)	0.911(0.757~1.096)
**5**	Ref	Ref	Ref
**6**	0.887(0.781~1.007)	0.928(0.817~1.054)	0.914(0.786~1.063)
**7**	0.817(0.725~0.921)**	0.867(0.769~0.977)*	0.839(0.728~0.967)*
**8**	0.741(0.656~0.838)**	0.804(0.711~0.909)**	0.758(0.654~0.878)**
**9**	0.812(0.719~0.918)**	0.910(0.805~1.029)	0.805(0.695~0.931)**
**Liking for sweet foods**			
**1**	1.046(0.893~1.225)	0.998(0.852~1.169)	1.023(0.846~1.238)
**2**	0.956(0.827~1.106)	0.933(0.806~1.079)	0.956(0.801~1.141)
**3**	0.793(0.692~0.908)**	0.783(0.684~0.898)**	0.842(0.715~0.990)*

**4**	0.942(0.832~1.066)	0.944(0.834~1.068)	0.962(0.828~1.117)
**5**	Ref	Ref	Ref
**6**	0.822(0.743~0.910)**	0.851(0.768~0.942)**	0.864(0.763~0.977)*
**7**	0.873(0.792~0.963)**	0.908(0.823~1.001)	0.928(0.825~1.045)
**8**	0.927(0.833~1.030)	0.973(0.875~1.082)	0.957(0.841~1.089)
**9**	1.044(0.935~1.165)	1.129(1.011~1.261)*	1.034(0.904~1.183)

Model 1 is a crude model, and Model 2 was adjusted for age and sex. We further adjusted ethnic background, BMI, smoking status, drinking status, education levels, average total household income before tax, MET minutes per week for activity, hypertension, diabetes, TG, total cholesterol, and HDL-C in Model 3. *, *P*-value <0.05,**, *P*-value <0.001

Table S14. Sensitive analysis of cox regression results for food preferences and depression (HR, 95%CI, and Significance) with excluding extreme HDL-C levels.

Variable	Model 1 HR (95%CI)	Model 2 HR (95%CI)	Model 3 HR (95%CI)
**Liking for bitter foods**			
**1**	1.361(1.136~1.631)**	1.270(1.057~1.525)*	1.236(0.987~1.547)
**2**	1.040(0.839~1.290)	1.000(0.806~1.240)	0.893(0.679~1.174)
**3**	0.975(0.791~1.202)	0.954(0.774~1.176)	0.938(0.724~1.215)
**4**	0.881(0.717~1.083)	0.881(0.717~1.083)	0.842(0.649~1.091)
**5**	Ref	Ref	Ref
**6**	0.797(0.651~0.976)*	0.817(0.667~1.000)	0.817(0.636~1.049)
**7**	0.739(0.600~0.911)*	0.765(0.620~0.943)*	0.706(0.539~0.924)*
**8**	0.807(0.609~1.068)	0.832(0.629~1.102)	1.022(0.739~1.412)
**9**	0.657(0.408~1.056)	0.682(0.424~1.098)	0.669(0.364~1.228)
**Liking for salty foods**			
**1**	1.165(0.932~1.457)	1.090(0.871~1.364)	0.961(0.737~1.252)
**2**	0.972(0.756~1.250)	0.927(0.721~1.192)	0.906(0.673~1.219)
**3**	0.878(0.685~1.126)	0.858(0.669~1.100)	0.891(0.673~1.179)
**4**	0.961(0.759~1.216)	0.952(0.752~1.204)	1.047(0.804~1.362)
**5**	Ref	Ref	Ref
**6**	0.844(0.677~1.053)	0.876(0.702~1.093)	0.854(0.662~1.102)
**7**	0.891(0.718~1.107)	0.944(0.760~1.172)	0.952(0.743~1.220)
**8**	0.706(0.534~0.933)*	0.752(0.569~0.995)*	0.739(0.537~1.017)
**9**	0.890(0.646~1.227)	0.955(0.692~1.318)	0.807(0.549~1.186)
**Liking for spicy foods**			
**1**	1.065(0.825~1.374)	1.029(0.797~1.329)	0.926(0.679~1.264)
**2**	0.875(0.642~1.191)	0.861(0.632~1.173)	0.701(0.474~1.037)
**3**	0.788(0.577~1.074)	0.784(0.575~1.069)	0.727(0.496~1.065)

**4**	0.624(0.454~0.856)*	0.633(0.461~0.870)*	0.652(0.449~0.947)*
**5**	Ref	Ref	Ref
**6**	0.715(0.564~0.908)*	0.757(0.596~0.960)*	0.732(0.550~0.975)*
**7**	0.591(0.472~0.741)**	0.632(0.504~0.793)**	0.605(0.461~0.794)**
**8**	0.571(0.454~0.719)**	0.631(0.501~0.795)**	0.587(0.443~0.776)**
**9**	0.662(0.528~0.830)**	0.759(0.604~0.954)*	0.633(0.479~0.836)*
**Liking for sweet foods**			
**1**	1.007(0.710~1.427)	0.965(0.681~1.368)	1.162(0.770~1.754)
**2**	1.113(0.830~1.491)	1.089(0.812~1.459)	1.265(0.887~1.805)
**3**	0.866(0.654~1.147)	0.855(0.645~1.132)	1.040(0.745~1.453)
**4**	0.965(0.744~1.251)	0.974(0.752~1.263)	1.052(0.760~1.457)
**5**	Ref	Ref	Ref
**6**	0.934(0.756~1.153)	0.969(0.785~1.196)	1.053(0.809~1.371)
**7**	0.980(0.799~1.202)	1.022(0.833~1.253)	1.095(0.849~1.414)
**8**	1.121(0.905~1.389)	1.183(0.955~1.467)	1.243(0.948~1.628)
**9**	1.231(0.985~1.539)	1.331(1.064~1.666)*	1.322(0.997~1.754)

Model 1 is a crude model, and Model 2 was adjusted for age and sex. We further adjusted ethnic background, BMI, smoking status, drinking status, education levels, average total household income before tax, MET minutes per week for activity, hypertension, diabetes, TG, total cholesterol, and HDL-C in Model 3. *, *P*-value <0.05,**, *P*-value <0.001

Table S15. Logistic regression results for food preferences and depressive symptoms in cross-sectional study.

**Variable**	**Model 1 OR (95%CI)**	**Model 2 OR (95%CI)**	**Model 3 OR (95%CI)**
**Liking for bitter foods**			
**1**	1.462 (1.383~1.545)**	1.439 (1.36~1.522)**	1.275 (1.08–1.503)**
**2**	1.099 (1.026~1.177)**	1.077 (1.004~1.155)*	0.957 (0.781–1.166)
**3**	1.095 (1.026~1.168)**	1.084 (1.015~1.157)*	0.948 (0.781–1.146)
**4**	1.046 (0.981~1.115)	1.034 (0.969~1.102)	0.992 (0.827–1.185)
**5**	ref.	ref.	ref.
**6**	0.957 (0.899~1.018)	0.934 (0.877~0.994)*	1.071 (0.905–1.265)
**7**	0.918 (0.861~0.979)**	0.901 (0.844~0.961)**	0.780 (0.646–0.939)**
**8**	0.949 (0.870~1.034)	0.939 (0.860~1.024)	1.147 (0.912–1.432)
**9**	1.283 (1.146~1.432)**	1.198 (1.068~1.339)**	1.137 (0.800–1.578)
**Liking for salty foods**			
**1**	1.045 (0.974~1.122)	1.048 (0.976~1.126)	1.182 (0.970–1.439)
**2**	0.894 (0.824~0.970)**	0.901 (0.829~0.978)*	1.180 (0.950–1.461)
**3**	0.930 (0.861~1.004)	0.947 (0.877~1.023)	0.964 (0.776–1.193)
**4**	0.909 (0.842~0.981)*	0.905 (0.838~0.977)*	0.925 (0.746–1.143)
**5**	ref.	ref.	ref.

**6**	0.979 (0.916~1.047)	0.942 (0.881~1.009)	1.021 (0.848–1.230)
**7**	1.034 (0.968~1.105)	0.988 (0.924~1.056)	1.187 (0.993–1.421)
**8**	1.152 (1.066~1.245)**	1.075 (0.994~1.162)	1.230 (0.999–1.513)
**9**	1.538 (1.413~1.673)**	1.351 (1.240~1.472)**	1.418 (1.116–1.794)**
**Liking for spicy foods**			
**1**	1.238 (1.140~1.345)**	1.228 (1.130~1.335)**	0.969 (0.752–1.248)
**2**	1.069 (0.966~1.181)	1.060 (0.957~1.173)	0.955 (0.708–1.279)
**3**	1.034 (0.937~1.139)	1.022 (0.926~1.128)	1.091 (0.827–1.436)
**4**	0.944 (0.857~1.040)	0.922 (0.835~1.014)	0.761 (0.569–1.012)
**5**	ref.	ref.	ref.
**6**	0.930 (0.860~1.005)	0.878 (0.811~0.950)	0.938 (0.752–1.175)
**7**	0.819 (0.761~0.882)**	0.782 (0.726~0.843)**	0.767 (0.621–0.952)*
**8**	0.813 (0.755~0.877)**	0.784 (0.727~0.846)**	0.794 (0.642–0.988)*
**9**	0.947 (0.880~1.020)	0.874 (0.811~0.942)**	0.801 (0.648–0.995)*
**Liking for sweet foods**			
**1**	1.006 (0.906~1.116)	1.036 (0.931~1.149)	1.044 (0.754–1.421)
**2**	0.911 (0.825~1.005)	0.929 (0.841~1.025)	1.007 (0.753–1.333)
**3**	0.911 (0.836~0.993)*	0.924 (0.847~1.007)	1.174 (0.924–1.487)
**4**	0.902 (0.830~0.980)*	0.898 (0.825~0.976)*	0.988 (0.776–1.253)
**5**	ref.	ref.	ref.
**6**	0.988 (0.926~1.054)	0.942 (0.883~1.006)	1.078 (0.894–1.303)
**7**	1.046 (0.983~1.114)	1.008 (0.947~1.074)	1.188 (0.993–1.426)
**8**	1.150 (1.076~1.229)**	1.096 (1.024~1.172)**	1.147 (0.944–1.397)
**9**	1.605 (1.504~1.713)**	1.440 (1.349~1.539)**	1.390 (1.144–1.693)**

Model 1 is a crude model, and Model 2 was adjusted for age and sex. We further adjusted ethnic background, BMI, smoking status, drinking status, education levels, average total household income before tax, MET minutes per week for activity, hypertension, diabetes, TG, total cholesterol, and HDL-C in Model 3. *, *P*-value <0.05,**, *P*-value <0.001

Table S16-23 were showed in supplementary table.xlsx

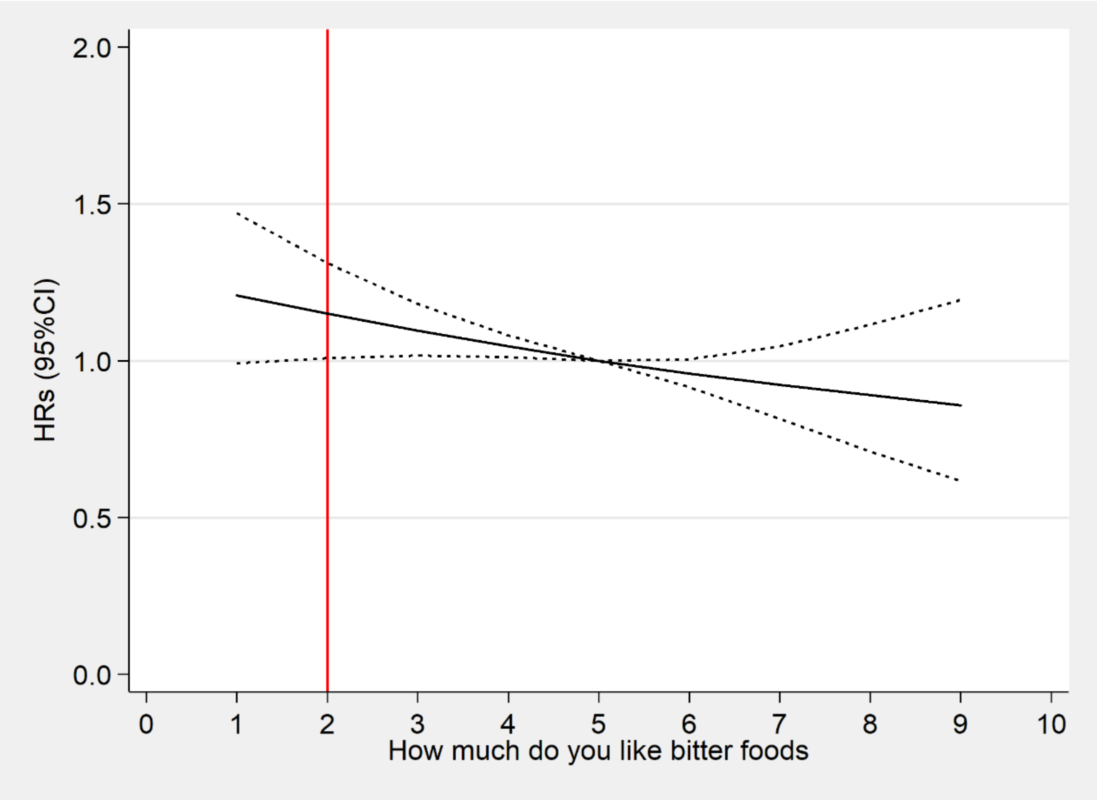

Figure S1. Restricted cubic spline for the risk to the extent of liking for bitter food. The associations were adjusted for age, sex, ethnic background, BMI, smoking status, drinking status, education levels, average total household income before tax, MET minutes per week for activity, hypertension, diabetes, TG, total cholesterol, and HDL-C. The solid and dashed lines represent the estimated HRs (Hazard Ratio) and their 95% confidence intervals.

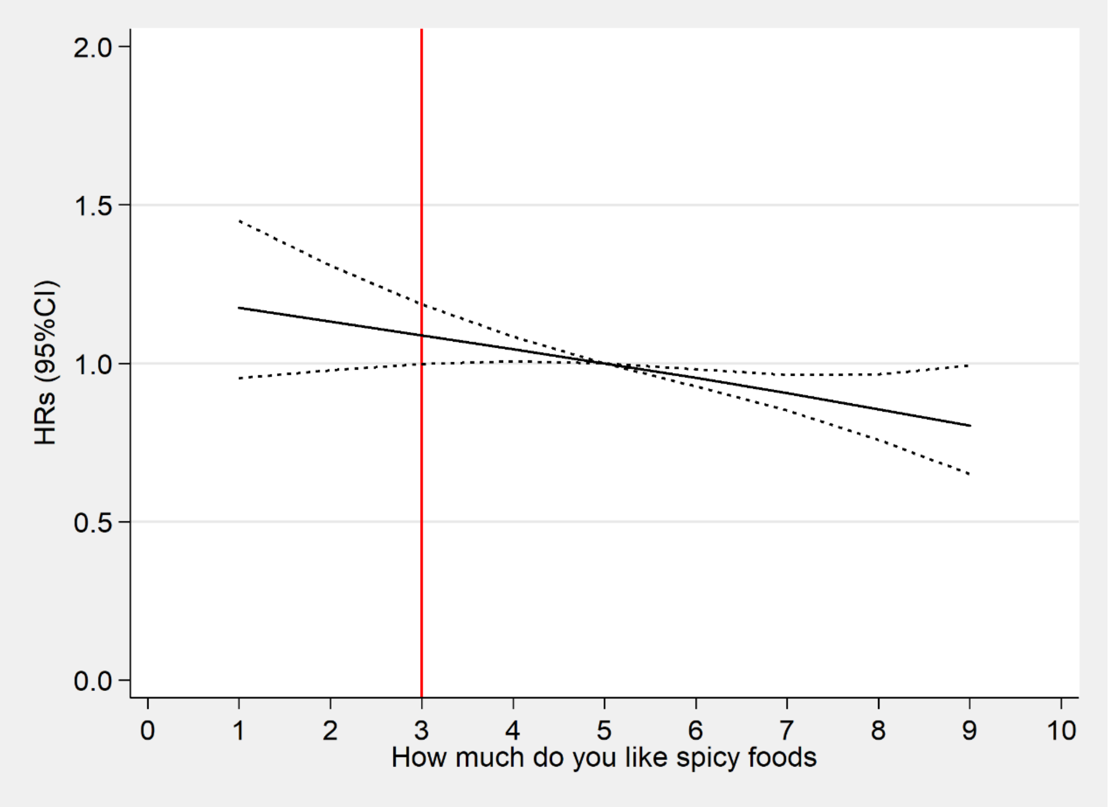

Figure S2. Restricted cubic spline for the risk to the extent of liking for spicy food. The associations were adjusted for age, sex, ethnic background, BMI, smoking status, drinking status, education levels, average total household income before tax, MET minutes per week for activity, hypertension, diabetes, TG, total cholesterol, and HDL-C. The solid and dashed lines represent the estimated HRs (Hazard Ratio) and their 95% confidence intervals.
